# Biomarker-directed targeted therapy plus durvalumab in advanced non-small-cell lung cancer: a phase 2 umbrella trial

**DOI:** 10.1038/s41591-024-02808-y

**Published:** 2024-02-13

**Authors:** Benjamin Besse, Elvire Pons-Tostivint, Keunchil Park, Sylvia Hartl, Patrick M. Forde, Maximilian J. Hochmair, Mark M. Awad, Michael Thomas, Glenwood Goss, Paul Wheatley-Price, Frances A. Shepherd, Marie Florescu, Parneet Cheema, Quincy S. C. Chu, Sang-We Kim, Daniel Morgensztern, Melissa L. Johnson, Sophie Cousin, Dong-Wan Kim, Mor T. Moskovitz, David Vicente, Boaz Aronson, Rosalind Hobson, Helen J. Ambrose, Sajan Khosla, Avinash Reddy, Deanna L. Russell, Mohamed Reda Keddar, James P. Conway, J. Carl Barrett, Emma Dean, Rakesh Kumar, Marlene Dressman, Philip J. Jewsbury, Sonia Iyer, Simon T. Barry, Jan Cosaert, John V. Heymach

**Affiliations:** 1 Institut Gustave Roussy, Paris-Saclay University, Villejuif, France; 2 Medical Oncology, Centre Hospitalier Universitaire Nantes, Nantes University, Nantes, France; 3 Samsung Medical Center, Sungkyunkwan University School of Medicine, Seoul, Republic of Korea; 4 Ludwig Boltzmann Institute for Lung Health, Clinic Penzing, Vienna, Austria; 5 Bloomberg-Kimmel Institute for Cancer Immunotherapy, Johns Hopkins University School of Medicine, Baltimore, MD USA; 6 Department of Respiratory and Critical Care Medicine, Karl Landsteiner Institute of Lung Research and Pulmonary Oncology, Klinik Floridsdorf, Vienna, Austria; 7 Dana-Farber Cancer Institute, Boston, MA USA; 8 Department of Thoracic Oncology, Thoraxklinik, Heidelberg University Hospital and National Center for Tumor Diseases (NCT), NCT Heidelberg, a partnership between DKFZ and Heidelberg University Hospital, Germany; Translational Lung Research Center Heidelberg (TLRC-H), Member of the German Center for Lung Research (DZL), Heidelberg, Germany; 9 The Ottawa Hospital Research Institute, University of Ottawa, Ottawa, Ontario Canada; 10 Department of Medical Oncology and Hematology, Princess Margaret Cancer Centre, University Health Network, Toronto, Ontario Canada; 11 Division of Hematology Oncology, Centre Hospitalier de l’Université de Montréal, Montreal, Quebec Canada; 12 William Osler Health System, University of Toronto, Toronto, Ontario Canada; 13 Cross Cancer Institute, Edmonton, Alberta Canada; 14 Department of Oncology, Asan Medical Center, Seoul, Republic of Korea; 15 Department of Medicine, Division of Medical Oncology, Washington University School of Medicine, St. Louis, MO USA; 16 Sarah Cannon Research Institute, Tennessee Oncology, Nashville, TN USA; 17 Department of Medical Oncology, Institut Bergonié, Regional Comprehensive Cancer Center, Bordeaux, France; 18 Seoul National University College of Medicine and Seoul National University Hospital, Seoul, Republic of Korea; 19 Institute of Oncology, Rambam Medical Center, Haifa, Israel; 20 Department of Medical Oncology, Hospital Universitario Virgen Macarena, Seville, Spain; 21 Oncology Early Global Development, AstraZeneca, Gaithersburg, MD USA; 22 Oncology Biometrics, AstraZeneca, Cambridge, UK; 23 Research and Early Development, Oncology R&D, AstraZeneca, Cambridge, UK; 24 Real-World Evidence, Oncology R&D, AstraZeneca, Cambridge, UK; 25 Oncology Data Science, Oncology R&D, AstraZeneca, Boston, MA USA; 26 Translational Medicine, Oncology R&D, AstraZeneca, Boston, MA USA; 27 Oncology Data Science, Research and Early Development, Oncology R&D, AstraZeneca, Cambridge, UK; 28 Oncology Data Science, Oncology R&D, AstraZeneca, Gaithersburg, MD USA; 29 Oncology R&D, AstraZeneca, Cambridge, UK; 30 Oncology R&D, AstraZeneca, Gaithersburg, MD USA; 31 Department of Thoracic/Head and Neck Medical Oncology, MD Anderson Cancer Center, Houston, TX USA; 32 Present Address: MD Anderson Cancer Center, Houston, TX USA; 33 Present Address: Sigmund Freud University, Vienna, Austria; 34 Present Address: Thoracic Cancer Service, Rabin Medical Center Davidoff Cancer Centre, Beilinson Campus, Petah Tikva, Israel

**Keywords:** Non-small-cell lung cancer, Tumour biomarkers

## Abstract

For patients with non-small-cell lung cancer (NSCLC) tumors without currently targetable molecular alterations, standard-of-care treatment is immunotherapy with anti-PD-(L)1 checkpoint inhibitors, alone or with platinum-doublet therapy. However, not all patients derive durable benefit and resistance to immune checkpoint blockade is common. Understanding mechanisms of resistance—which can include defects in DNA damage response and repair pathways, alterations or functional mutations in *STK11*/LKB1, alterations in antigen-presentation pathways, and immunosuppressive cellular subsets within the tumor microenvironment—and developing effective therapies to overcome them, remains an unmet need. Here the phase 2 umbrella HUDSON study evaluated rational combination regimens for advanced NSCLC following failure of anti-PD-(L)1-containing immunotherapy and platinum-doublet therapy. A total of 268 patients received durvalumab (anti-PD-L1 monoclonal antibody)–ceralasertib (ATR kinase inhibitor), durvalumab–olaparib (PARP inhibitor), durvalumab–danvatirsen (STAT3 antisense oligonucleotide) or durvalumab–oleclumab (anti-CD73 monoclonal antibody). Greatest clinical benefit was observed with durvalumab–ceralasertib; objective response rate (primary outcome) was 13.9% (11/79) versus 2.6% (5/189) with other regimens, pooled, median progression-free survival (secondary outcome) was 5.8 (80% confidence interval 4.6–7.4) versus 2.7 (1.8–2.8) months, and median overall survival (secondary outcome) was 17.4 (14.1–20.3) versus 9.4 (7.5–10.6) months. Benefit with durvalumab–ceralasertib was consistent across known immunotherapy-refractory subgroups. In *ATM*-altered patients hypothesized to harbor vulnerability to ATR inhibition, objective response rate was 26.1% (6/23) and median progression-free survival/median overall survival were 8.4/22.8 months. Durvalumab–ceralasertib safety/tolerability profile was manageable. Biomarker analyses suggested that anti-PD-L1/ATR inhibition induced immune changes that reinvigorated antitumor immunity. Durvalumab–ceralasertib is under further investigation in immunotherapy-refractory NSCLC.

ClinicalTrials.gov identifier: NCT03334617

## Abstract (web summary)

In the phase 2 HUDSON study, patients with advanced non-small-cell lung cancer received anti-PD-L1 combined with biomarker-guided therapy targeting ATR kinase, PARP, STAT3 or CD73, leading to encouraging clinical benefit in response to combination of the ATR kinase inhibitor ceralasertib with durvalumab.

## Main

Patients with advanced non-small-cell lung cancer (NSCLC) receive initial therapy based on molecular classification of their disease^1^. For NSCLC tumors without currently targetable molecular alterations^2^ (>50% of adenocarcinomas^3^ and >95% of squamous cell carcinomas^4^), standard-of-care treatment is immunotherapy with anti-programmed death (ligand)-1 (PD-(L)1) checkpoint inhibitors, alone or with platinum-doublet therapy^1^, which offers long-term disease control and overall survival (OS) benefit for some patients^5–12^. Tumor-intrinsic factors and the tumor microenvironment (TME) influence response to immune checkpoint blockade (ICB)^13–16^, including genomic features associated with differential response to ICB. Although high PD-L1 expression levels^7,17^ and tumor mutational burden (TMB)^18^ have emerged as important biomarkers, not all patients derive durable benefit, and resistance to ICB is common. Clinical resistance to ICB is complex and can present at various time points during treatment; primary resistance is defined as a best response of disease progression or stable disease lasting <6 months (24 weeks) and acquired resistance as progression following a response or stable disease lasting ≥6 months^13,19–21^. Biological mechanisms that render patients with NSCLC recalcitrant to ICB include alterations or functional mutations resulting in the inactivation of the tumor suppressor gene *STK11*/LKB1 and a consequent reduction in tumor-infiltrating lymphocytes^22–25^, inactivating mutations of *KEAP1* (refs. ^26,27^), low expression of PD-L1, and alterations in antigen-presentation pathways. Furthermore, immunosuppressive cellular subsets within the TME such as myeloid-derived suppressor cells (MDSCs), T regulatory cells and others influence responses to ICB^16^. Understanding these resistance mechanisms, and developing effective therapies to overcome them, remains a critical unmet need as there are currently no immunotherapy-based regimens approved for patients with NSCLC who have progressed after initial ICB.

A major challenge in this setting is our poor understanding of the key immunotherapy-resistance mechanisms in primary and acquired resistance. While genotype/biomarker-directed targeted therapy is relatively well understood and can offer durable efficacy in NSCLC^28–30^, less is understood about biomarker subgroups in the immunotherapy setting^16^ that may be associated with reduced efficacy. The ongoing, open-label, multicenter, nonrandomized, modular, phase 2 umbrella HUDSON study (NCT03334617) was designed to build a comprehensive understanding of key features in patients and their tumors associated with disease progression and to differentiate tumor characteristics and treatment activity in primary and acquired resistance phenotypes. Rational combination therapies were selected for investigation in HUDSON based on targeting proposed mechanisms of immunosuppression with the aim of reinvigorating immune-mediated antitumor activity.

Mismatch repair deficiency is associated with greater benefit from immunotherapies^31–34^; HUDSON therefore evaluated inhibitors of DNA damage response (DDR) and repair pathways in combination with the anti-PD-L1 monoclonal antibody durvalumab. DDR pathway defects generate immunogenic neoantigens that are recognized and targeted by T cells^32,35,36^. Inhibition of poly-(ADP-ribose) polymerase (PARP)^37–39^ and ataxia telangiectasia and Rad3-related (ATR) protein kinase^40,41^ represent two key approaches for targeting DDR defects, and HUDSON evaluated both PARP and ATR inhibitors in combination with durvalumab. Another approach may be to target immunosuppression in the TME and resistance lymph nodes. For example, signal transducer and activator of transcription 3 (STAT3) signaling results in production of tumor-promoting cytokines (by myeloid cells in the TME) and inhibition of antitumor cytokines, shifting the TME toward an immunosuppressive state^42^. Targeting STAT3 may help reverse immunosuppression by myeloid cells and directly attack tumor cells (TCs)^43^. Similarly, targeting anti-5′-nucleotidase cluster of differentiation 73 (CD73) may help reverse the immunosuppressive TME by increasing the activity of CD8-positive effector cells, activating macrophages and reducing both MDSCs and regulatory T lymphocytes^44,45^.

In this Article, we report the first clinical efficacy, safety, and translational data from four modules of HUDSON in patients who have received a platinum-doublet regimen and progressed on anti-PD-(L)1-based therapy. Treatments received in these modules were durvalumab plus ceralasertib (ATR kinase inhibitor), olaparib (PARP inhibitor), danvatirsen (STAT3-targeting antisense oligonucleotide) or oleclumab (anti-CD73 monoclonal antibody). Patients underwent tumor molecular profiling at screening and were assigned to either a biomarker-matched cohort (durvalumab–ceralasertib: *ATM* altered; durvalumab–olaparib: homologous recombination repair (HRR) altered, *STK11*/*LKB1* altered; durvalumab–oleclumab: high CD73 expression) or a biomarker-nonmatched group. Patients in the latter group were further stratified into primary or acquired resistance cohorts (see ‘Study design’ in Methods). Multi-omic longitudinal peripheral biomarker profiling was used for hypothesis-generating exploratory analyses of possible underlying mechanisms of action driving treatment outcomes.

## Results

### Patients

**Fig. 1:**
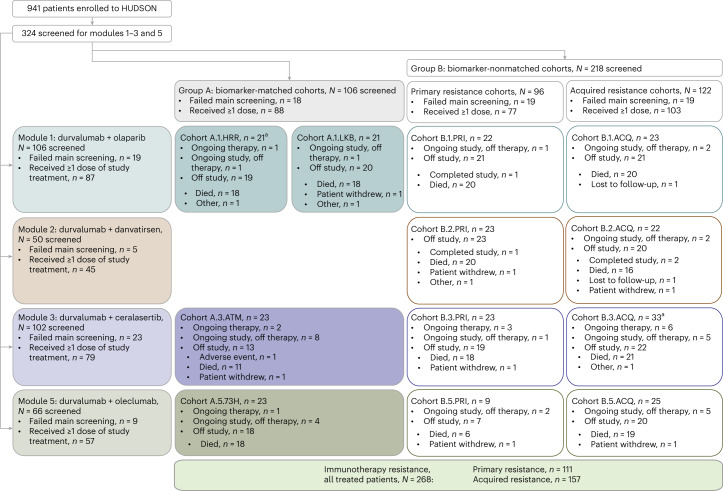
CONSORT diagram of patient screening and disposition in HUDSON. Based on tumor molecular profiling, patients were assigned to either biomarker-matched (Group A) or non-matched cohorts (Group B), which included patients with primary and acquired resistance determined by their initial response to prior immunotherapy-containing regimens. Patients were then treated with one of four durvalumab-based combination regimens (Modules 1–4).

Between 26 January 2018 and 26 April 2022, 941 patients were screened for enrollment in HUDSON, including 324 for the four modules reported herein (Fig. 1); of these, 268 patients received treatment, 88 within biomarker-matched cohorts and 77 and 103 within primary and acquired resistance biomarker-nonmatched cohorts, respectively. Overall, 79 patients received durvalumab–ceralasertib and 189 received the other regimens, including 87, 45 and 57 who received durvalumab–olaparib, durvalumab–danvatirsen and durvalumab–oleclumab, respectively. Patient demographics and disease characteristics were similar between treatment modules (Table 1).

**Table 1:** Patient baseline characteristics according to combination regimen received

Characteristic	Durvalumab–ceralasertib, *n* = 79	Durvalumab plus other agents			
		All, pooled, *n* = 189	Olaparib, *n* = 87	Danvatirsen, *n* = 45	Oleclumab, *n* = 57
Age, median (range), years	63.0 (42–80)	64.0 (35–85)	63.0 (35–85)	65.0 (39–80)	64.0 (37–79)
Age <65 years, *n* (%)	45 (57.0)	102 (54.0)	49 (56.3)	21 (46.7)	32 (56.1)
Age ≥65 years, *n* (%)	34 (43.0)	87 (46.0)	38 (43.7)	24 (53.3)	25 (43.9)
Male sex, *n* (%)^a^	52 (65.8)	103 (54.5)	50 (57.5)	23 (51.1)	30 (52.6)
Race, *n* (%)^b^	*n* = 77	*n* = 187	*n* = 86	*n* = 44	*n* = 57
White	47 (61.0)	124 (66.3)	58 (67.4)	29 (65.9)	37 (64.9)
Asian	9 (11.7)	38 (20.3)	23 (26.7)	7 (15.9)	8 (14.0)
Black or African American	3 (3.9)	4 (2.1)	0	4 (9.1)	0
Native Hawaiian or Other Pacific Islander	0	1 (0.5)	0	0	1 (1.8)
Other^c^	18 (23.4)	20 (10.7)	5 (5.8)	4 (9.1)	11 (19.3)
ECOG PS, *n* (%)^b^	*n* = 79	*n* = 188	*n* = 87	*n* = 44	*n* = 57
0	28 (35.4)	64 (34.0)	20 (23.0)	21 (47.7)	23 (40.4)
1	51 (64.6)	123 (65.4)	66 (75.9)	23 (52.3)	34 (59.6)
2^d^	0	1 (0.5)	1 (1.1)	0	0
Histology, *n* (%)					
Adenocarcinoma	55 (69.6)	131 (69.3)	62 (71.3)	31 (68.9)	38 (66.7)
Squamous cell carcinoma	19 (24.1)	43 (22.8)	18 (20.7)	12 (26.7)	13 (22.8)
Large-cell carcinoma (NOS)	2 (2.5)	6 (3.2)	4 (4.6)	1 (2.2)	1 (1.8)
Other	3 (3.8)	9 (4.8)	3 (3.4)	1 (2.2)	5 (8.8)
Time from diagnosis, *n* (%)^b^	*n* = 79	*n* = 184	*n* = 85	*n* = 43	*n* = 56
≤12 months	13 (16.6)	44 (23.9)	19 (22.4)	14 (32.6)	11 (19.6)
>12 months	66 (83.5)	140 (76.1)	66 (77.6)	29 (67.4)	45 (80.4)
Group and cohort, *n* (%)^b^					
Group A, biomarker matched	23 (29.1)	65 (34.4)	42 (48.3)	0	23 (40.4)
Group B, biomarker nonmatched	56 (70.9)	124 (65.6)	45 (51.7)	45 (100)	34 (59.6)
Primary resistance cohort	23 (29.1)	54 (28.6)	22 (25.3)	23 (51.1)	9 (15.8)
Acquired resistance cohort	33 (41.8)	70 (37.0)	23 (26.4)	22 (48.9)	25 (43.9)
Resistance classification (pooled groups A and B)					
Primary resistance	30 (38.0)	81 (42.9)	39 (44.8)	23 (51.1)	19 (33.3)
Acquired resistance	49 (62.0)	108 (57.1)	48 (55.2)	22 (48.9)	38 (66.7)
Disease classification, *n* (%)^b^	*n* = 79	*n* = 188	*n* = 87	*n* = 44	*n* = 57
Metastatic	77 (97.5)	184 (97.9)	87 (100)	43 (97.7)	54 (94.7)
Locally advanced	2 (2.5)	4 (2.1)	0	1 (2.3)	3 (5.3)
Metastatic sites, *n* (%)					
≤2	31 (39.2)	112 (59.3)	40 (46.0)	39 (86.7)	33 (57.9)
≥3	48 (60.8)	77 (40.7)	47 (54.0)	6 (13.3)	24 (42.1)
Metastatic site location, *n* (%)					
Bone and locomotor	25 (31.6)	57 (30.1)	31 (35.6)	7 (15.6)	19 (33.3)
Adrenal gland	15 (19.0)	37 (19.6)	19 (21.8)	8 (17.8)	10 (17.5)
Brain/CNS and/or other CNS	12 (15.2)	37 (19.6)	21 (24.1)	6 (13.3)	10 (17.5)
Liver and/or hepatic (including gall bladder)	14 (17.7)	36 (19.0)	15 (17.2)	4 (8.9)	17 (29.8)
PD-L1 status, locally assessed, *n* (%)					
Positive (TC ≥1%)	42 (53.2)	85 (45.0)	27 (31.0)	24 (53.3)	34 (59.6)
1–49%	25 (31.6)	46 (24.3)	14 (16.1)	14 (31.1)	18 (31.6)
≥50%	17 (21.5)	39 (20.6)	13 (14.9)	10 (22.2)	16 (28.1)
Negative (TC <1%)	21 (26.6)	30 (15.9)	16 (18.4)	6 (13.3)	8 (14.0)
Unknown	16 (20.3)	73 (38.6)	43 (49.4)	15 (33.3)	15 (26.3)
Missing	0	1 (0.5)	1 (1.1)	0	0
Prior regimens, *n* (%)					
1	15 (19.0)	22 (11.6)	12 (13.8)	3 (6.7)	7 (12.3)
2	27 (34.2)	85 (45.0)	39 (44.8)	22 (48.9)	24 (42.1)
3	22 (27.8)	47 (24.9)	20 (23.0)	13 (28.9)	13 (22.8)
≥4	15 (19.0)	35 (18.5)	16 (18.4)	7 (15.6)	12 (21.1)
Prior immunotherapies, *n* (%)					
1	77 (97.5)	188 (99.5)	87 (100)	44 (97.8)	57 (100)
2	2 (2.5)	1 (0.5)	0	1 (2.2)	0
Prior anti-PD-(L)1 immunotherapy, *n* (%)					
Nivolumab	36 (45.6)	93 (49.2)	51 (58.6)	19 (42.2)	23 (40.4)
Pembrolizumab	33 (41.8)	52 (27.5)	20 (23.0)	15 (33.3)	17 (29.8)
Atezolizumab	7 (8.9)	26 (13.8)	9 (10.3)	8 (17.8)	9 (15.8)
Durvalumab	1 (1.3)	17 (9.0)	6 (6.9)	4 (8.9)	7 (12.3)
Cemiplimab	0	1 (0.5)	0	0	1 (1.8)
Prior anti-CTLA4 immunotherapy, *n* (%)					
Tremelimumab	1 (1.3)	5 (2.6)	3 (3.4)	1 (2.2)	1 (1.8)
Ipilimumab	0	2 (1.1)	2 (2.3)	0	0
Best response on prior immunotherapy, *n* (%)					
Complete response	0	2 (1.1)	0	1 (2.2)	1 (1.8)
Partial response	17 (21.5)	54 (28.6)	25 (28.7)	12 (26.7)	17 (29.8)
Stable disease	40 (50.6)	64 (33.9)	29 (33.3)	8 (17.8)	27 (47.4)
Stable disease, biomarker-matched patients	11 (13.9)	27 (14.3)	15 (17.2)	0	12 (21.1)
Stable disease, biomarker-nonmatched patients	29 (36.7)	37 (19.6)	14 (16.1)	8 (17.8)	15 (26.3)
Progressive disease	16 (20.3)	57 (30.2)	24 (27.6)	22 (48.9)	11 (19.3)
Nonevaluable	3 (3.8)	10 (5.3)	7 (8.0)	2 (4.4)	1 (1.8)
Not applicable	1 (1.3)	1 (0.5)	1 (1.1)	0	0
Time from prior immunotherapy	*n* = 77	*n* = 188	*n* = 86	*n* = 45	*n* = 57
Median, months (range)	3.9 (0.7–31.4)	3.2 (0.7–50.1)	2.5 (0.7–50.1)	3.7 (0.8–30.3)	3.7 (0.8–24.7)
Prior immunotherapy and resistance classification					
Primary resistance, *n* (%)	29 (36.7)	80 (42.3)	38 (43.7)	23 (51.1)	19 (33.3)
Prior monotherapy	22 (27.8)	63 (33.3)	31 (35.6)	21 (46.7)	11 (19.3)
Prior combination	5 (6.3)	16 (8.5)	7 (8.0)	2 (4.4)	7 (12.3)
Both monotherapy and combination	2 (2.5)	1 (0.5)	0	0	1 (1.8)
Acquired resistance, *n* (%)	48 (60.8)	108 (57.1)	48 (55.2)	22 (48.9)	38 (66.7)
Prior monotherapy	36 (45.6)	85 (45.0)	36 (41.4)	18 (40.0)	31 (54.4)
Prior combination	7 (8.9)	18 (9.5)	10 (11.5)	4 (8.9)	4 (7.0)
Both monotherapy and combination	5 (6.3)	5 (2.6)	2 (2.3)	0	3 (5.3)
Not available, *n* (%)	2 (2.5)	1 (0.5)	1 (1.1)	0	0
Prior platinum-based therapies, *n* (%)					
1	62 (78.5)	154 (81.5)	70 (80.5)	39 (86.7)	45 (78.9)
2	15 (19.0)	28 (14.8)	14 (16.1)	4 (8.9)	10 (17.5)
≥3	2 (2.5)	7 (3.7)	3 (3.4)	2 (4.4)	2 (3.5)
Smoking status, *n* (%)					
Never	9 (11.4)	28 (14.8)	18 (20.7)	5 (11.1)	5 (8.8)
Current	18 (22.8)	23 (12.2)	6 (6.9)	6 (13.3)	11 (19.3)
Former	52 (65.8)	138 (73.0)	63 (72.4)	34 (75.6)	41 (71.9)

^a^Sex as recorded by investigator in the case report form.

^b^For parameters for which data are missing, the numbers of patients with data are indicated for each regimen and are used as the denominators for calculating percentages.

^c^Recorded as ‘Other’ in the case report form.

^d^Patient did not meet the eligibility criterion for ECOG PS of 0–1.

CNS, central nervous system; ECOG PS, Eastern Cooperative Oncology Group performance status; NOS, not otherwise specified.

### Notable clinical efficacy with ceralasertib–durvalumab

Efficacy was evaluated with the four rational combinations to determine whether targeting specific pathways potentially associated with resistance could improve outcomes. Objective response rate (ORR), the primary endpoint, was 13.9% (*n* = 11/79) with durvalumab–ceralasertib, whereas the pooled ORR across the other treatment modules was 2.6% (*n* = 5/189) (Extended Data Table 1). All objective responses were confirmed partial responses (see Supplementary Table 1 for details of prior treatment in responding patients). Disease control rates at 12 and 24 weeks, respectively, were 50.6% and 35.4% with durvalumab–ceralasertib and 32.3% and 15.9% with the pooled other regimens (Extended Data Table 1).

**Fig. 2:**
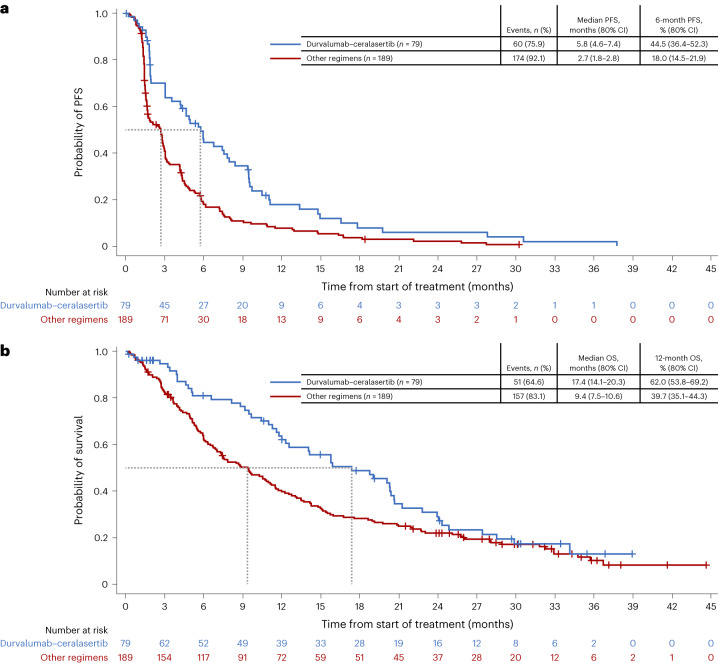
PFS and OS with durvalumab–ceralasertib and with durvalumab plus olaparib, danvatirsen or oleclumab in HUDSON. **a**,**b**, Kaplan–Meier analysis of PFS (**a**) and OS (**b**) among all patients who received durvalumab–ceralasertib or who received durvalumab plus olaparib, danvatirsen or oleclumab on HUDSON.

Longer progression-free survival (PFS) and OS were seen with durvalumab–ceralasertib compared with the pooled other regimens, with a median PFS of 5.8 (80% confidence interval (CI) 4.6–7.4) and 2.7 (1.8–2.8) months, respectively, and a median OS of 17.4 (14.1–20.3) and 9.4 (7.5–10.6) months, respectively (Fig. 2a, b and Extended Data Table 1), after median follow-up times in censored patients of 8.3 and 28.2 months.

### Durvalumab–ceralasertib in *ATM* biomarker-matched patients

**Extended Data Fig. 1:**
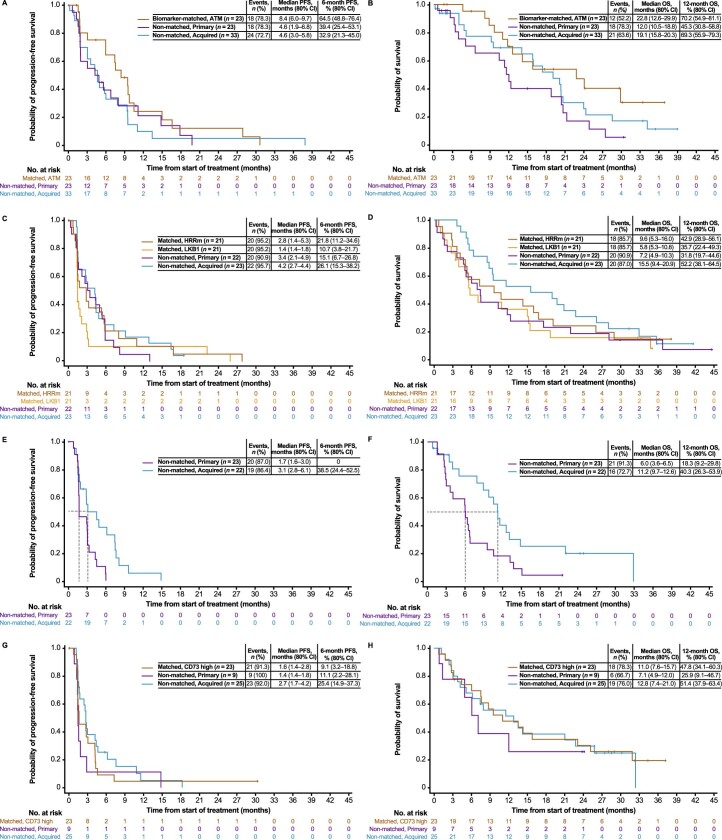
PFS and OS by treatment cohort. PFS and OS with **a, b**, durvalumab-ceralasertib (median OS not yet mature in *ATM* biomarker-matched cohort), **c, d**, durvalumab-olaparib, **e, f**, durvalumab-danvatirsen, **g, h**, durvalumab-oleclumab.

In patients with genetic or immunosuppressive biomarkers of relevance, the mechanisms of action of the partner drugs were investigated to determine the efficacy and specific sensitivity of durvalumab-based combinations in these settings. Patient demographics and baseline disease characteristics in the biomarker-matched and biomarker-nonmatched groups are summarized in Supplementary Table 2. Responses and outcomes by treatment module and in individual biomarker-matched and primary and acquired biomarker-nonmatched cohorts are summarized in Extended Data Table 2 for durvalumab–ceralasertib and in Supplementary Tables 3–5 for durvalumab–olaparib, durvalumab–danvatirsen and durvalumab–oleclumab. Kaplan–Meier analyses of PFS and OS by cohort are shown in Extended Data Fig. 1.

*ATM* alterations confer ATR dependency in tumors. We observed an ORR of 26.1% (*n* = 6/23) with durvalumab–ceralasertib in the *ATM*-altered biomarker-matched cohort, higher than the ORRs of 13.0% (*n* = 3/23) and 6.1% (*n* = 2/33) in the primary and acquired resistance biomarker-nonmatched cohorts, respectively (Extended Data Table 2), suggesting specific benefit of ATR inhibition in patients with *ATM* alterations. Additionally, PFS (median 8.4 versus 4.6 versus 4.6 months) and OS (median 22.8 versus 12.0 versus 19.1 months) appeared longer in the *ATM*-altered biomarker-matched cohort versus the primary and acquired resistance cohorts, respectively (Extended Data Fig. 1a,b). Notably, while OS appeared longer in the acquired versus primary resistance cohorts, PFS was similar.

In contrast, PARP inhibition has been successful in targeting tumors with DDR defects in other solid tumors, and preclinical data suggest that *STK11*/LKB1 alterations may be associated with enhanced PARP inhibitor sensitivity^25,46^. However, ORR with durvalumab–olaparib was 4.6%, including 9.5% and 4.8% in the HRRm and *STK11*/LKB1 biomarker-matched cohorts, suggesting limited vulnerability from targeting these mutations, and 0% and 4.3% in the primary and acquired resistance cohorts (Supplementary Table 3). Similarly, no objective responses were seen with durvalumab–danvatirsen (Supplementary Table 4), indicating that reprogramming macrophages with STAT3 inhibition using an antisense oligonucleotide does not reverse myeloid-mediated resistance, even in the context of *STK11* alterations, which have been associated with suppression of mononuclear and polymorphonuclear MDSCs^22,26^. One partial response (1.8%) was seen with durvalumab–oleclumab (Supplementary Table 5), suggesting that reducing adenosine levels through CD73 inhibition is insufficient to reverse CD73-dependent immunosuppression. PFS and OS with durvalumab–olaparib (Extended Data Fig. 1c,d) and durvalumab–oleclumab (Extended Data Fig. 1g,h) appeared similar or shorter in the biomarker-matched versus biomarker-nonmatched cohorts, with PFS and OS with each regimen also appearing shorter in the biomarker-nonmatched primary versus acquired resistance cohorts (Extended Data Fig. 1c–h).

### Treatment exposure and safety

Mean durvalumab treatment duration was longer with durvalumab–ceralasertib (8.7 months) than the other regimens (5.1 months), and this was reflected across cohorts (Extended Data Table 3). With durvalumab–ceralasertib and the other regimens, similar overall incidences of treatment-emergent adverse events (TEAEs; 93.7% and 89.9%), grade ≥3 TEAEs (44.3% and 51.3%), treatment-related TEAEs (TRAEs; 75.9% and 71.4%), grade ≥3 TRAEs (20.3% and 30.2%) and serious AEs (SAEs; 36.7% and 34.4%) were reported (Extended Data Table 4). Incidences of treatment-related SAEs (12.7% and 9.0%) and treatment discontinuation due to TRAEs (7.6% and 10.1%) were low and similar with durvalumab–ceralasertib and the other regimens, respectively. Two patients (2.5%) receiving durvalumab–ceralasertib (pneumonia and myocardial infarction, each *n* = 1) and five patients (2.6%) receiving the other regimens (cor pulmonale, dyspnea, sepsis, pneumonia aspiration and renal artery thrombosis, each *n* = 1), died due to a TEAE; none was considered related to treatment. Safety profiles by regimen, overall and by cohort, are summarized in Supplementary Tables 6–9.

Among Medical Dictionary for Regulatory Activities (MedDRA) System Organ Class (SOC) categories, the incidences of gastrointestinal disorders (58.2% and 34.9%), metabolism and nutrition disorders (25.3% and 10.1%) and nervous system disorders (19.0% and 4.8%) showed >10 percentage point differences between durvalumab–ceralasertib and the other regimens, pooled; of the individual TRAEs occurring in ≥10% of patients treated with durvalumab–ceralasertib or other regimens, nausea (50.6% and 22.2%), vomiting (27.8% and 11.1%) and decreased appetite (21.5% and 7.4%) were the only TRAEs for which there was a >10 percentage point difference between groups (Extended Data Table 4). Incidences of TRAEs by MedDRA SOC and of individual TRAEs, and common grade ≥3 TRAEs by individual regimen, are summarized in Extended Data Tables 5 and 6, respectively. With durvalumab–ceralasertib, the only grade ≥3 TRAE with an incidence of ≥5% was thrombocytopenia (5.1%), while with durvalumab–olaparib anemia was reported in 13.8% of patients, and with durvalumab–danvatirsen increased alanine aminotransferase was reported in 11.1% and thrombocytopenia in 8.9% of patients (Extended Data Table 6).

### Distinct features delineate patients with ICB-recalcitrant NSCLC

In an exploratory post-hoc analysis to examine clinical and molecular biomarker characteristics associated with recalcitrance to ICB, we measured TME inflammation status using a validated 18-gene tumor inflammation signature (TIS)^47,48^ in subgroups with known association with poor immunotherapy response among patients with evaluable prescreening tumor biopsy suitable for bulk-RNA sequencing who received durvalumab plus olaparib, danvatirsen or oleclumab (Supplementary Table 10). This composite signature, which reflects the state, function and phenotype of the TME and is a widely used predictive biomarker for benefit with ICB therapy^47,49,50^, was used to characterize TME immune and inflammation status in patient subgroups in which clinical data for durvalumab–ceralasertib and durvalumab plus olaparib, danvatirsen or oleclumab were evaluated.

**Fig. 3:**
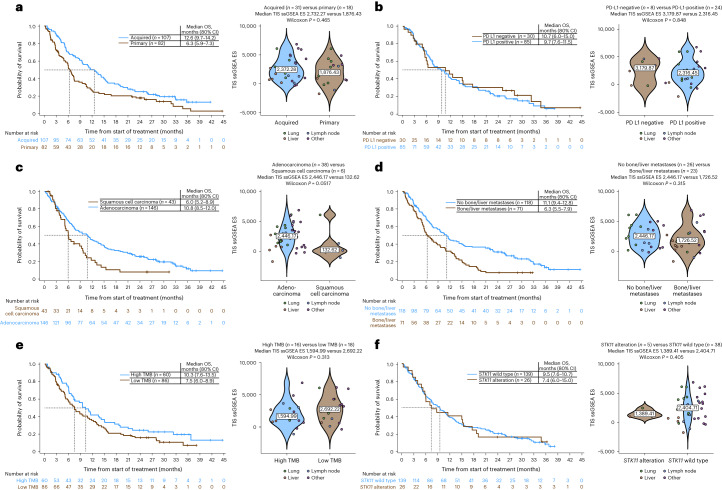
OS and gene expression profiling in subgroups known to be associated with poor immunotherapy response among patients who received durvalumab plus olaparib, danvatirsen or oleclumab. **a**–**f**, Kaplan–Meier distributions of OS (left) and TIS ssGSEA enrichment score (ES) distributions (violin plots, right) comparing patients with primary or acquired resistance (**a**), PD-L1-negative or PD-L1-positive status (**b**), squamous cell carcinoma or adenocarcinoma histology (**c**), with or without bone/liver metastases (**d**), high or low TMB (**e**) and *STK11*-altered or *STK11*-wild-type tumors (**f**). Median OS, 80% CIs and numbers of patients at risk are shown in each Kaplan–Meier plot. Violin plots show data from individual patients (dots), color-coded by site of sample, the median value (text box) and the probability density distribution smoothed using a kernel density estimator (shaded areas). ssGSEA ES distributions were compared using two-sided Wilcoxon rank-sum tests, with no correction for multiplicity of testing; associated *P* values and the number of tumor samples in each group are reported for each comparison.

**Extended Data Fig. 2:**
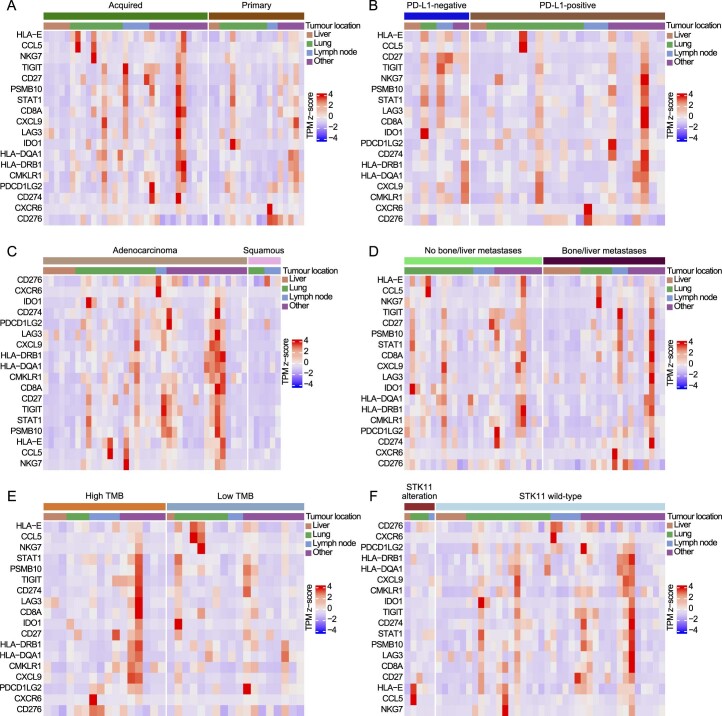
Gene expression profiling in subgroups with known association with poor immunotherapy response among patients who received durvalumab plus olaparib, danvatirsen or oleclumab. Gene-wise transcript-per-million (TPM) z-score heatmaps for the 18 genes comprising the tumour inflammation signature (TIS) in tumour samples from patients with **a**, primary or acquired resistance to prior immune checkpoint blockade therapy (*n* = 18, *n* = 31; overall prevalence: *n* = 81 [43.1%], *n* = 107 [56.9%]), **b**, PD-L1-positive or PD-L1-negative tumours (*n* = 16, *n* = 18; overall prevalence: *n* = 73 [58.9%], *n* = 51 [41.1%]), **c**, adenocarcinoma or squamous cell histology (*n* = 38, *n* = 6; overall prevalence: *n* = 130 [75.1%], *n* = 43 [24.9%]), **d**, with or without bone/liver metastases (*n* = 23, *n* = 26; overall prevalence: *n* = 76 [40.4%], *n* = 112 [59.6%]), **e**, high or low TMB (*n* = 16, *n* = 18; overall prevalence: *n* = 60 [41.1%], *n* = 86 [58.9%]) and **f**, *STK11*-altered or *STK11*-wild-type tumours (*n* = 5, *n* = 38; overall prevalence: *n* = 26 [14.9%], *n* = 148 [85.1%]). Tumour location and histology are annotated.

TIS scores (with pooled OS data among all patients who received durvalumab plus olaparib, danvatirsen or oleclumab, for context) are shown in Fig. 3. OS was numerically longer and TIS score higher among patients with acquired versus primary resistance, with multiple markers of inflammation including lymphocyte-activation gene 3 (LAG3), T cell immunoreceptor with immunoglobulin and immunoreceptor tyrosine-based inhibitory motif (ITIM) domains (TIGIT) and nonclassical human leukocyte antigen (HLA) molecule HLA-E more highly expressed in those with acquired resistance to prior immunotherapy (Fig. 3a and Extended Data Fig. 2a). Analysis according to PD-(L)1 TC expression status (<1%, negative, versus ≥1%, positive) showed similar outcomes in both groups and a similar TIS score (Fig. 3b) but with elevated expression of some inflammation markers in some PD-(L)1-positive tumors (Extended Data Fig. 2b). OS appeared longer and TIS score was higher (*P* = 0.0517) in patients with adenocarcinoma versus squamous cell histology, with most markers of inflammation more highly expressed in adenocarcinomas (Fig. 3c). Similarly, the presence of bone and/or liver metastases was associated with shorter OS; however, there was no difference in TIS score between those with or without metastases, albeit sites of biopsy differed and may have impacted the findings (Fig. 3d). Outcomes and TIS score were broadly similar in subgroups with high or low TMB, and altered or wild-type *STK11* (Fig. 3e,f). Heat maps showing expression of the 18 genes in the TIS in the above subgroups are shown in Extended Data Fig. 2.

### Durvalumab–ceralasertib broadly active in patient subgroups

**Fig. 4:**
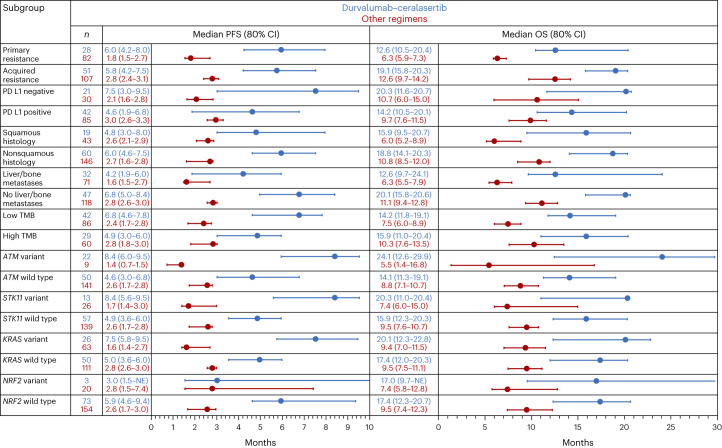
PFS and OS with durvalumab–ceralasertib and with durvalumab plus olaparib, danvatirsen or oleclumab in HUDSON in patient subgroups. Figure shows medians and 80% CI per Kaplan–Meier methodology.

**Extended Data Fig. 3:**
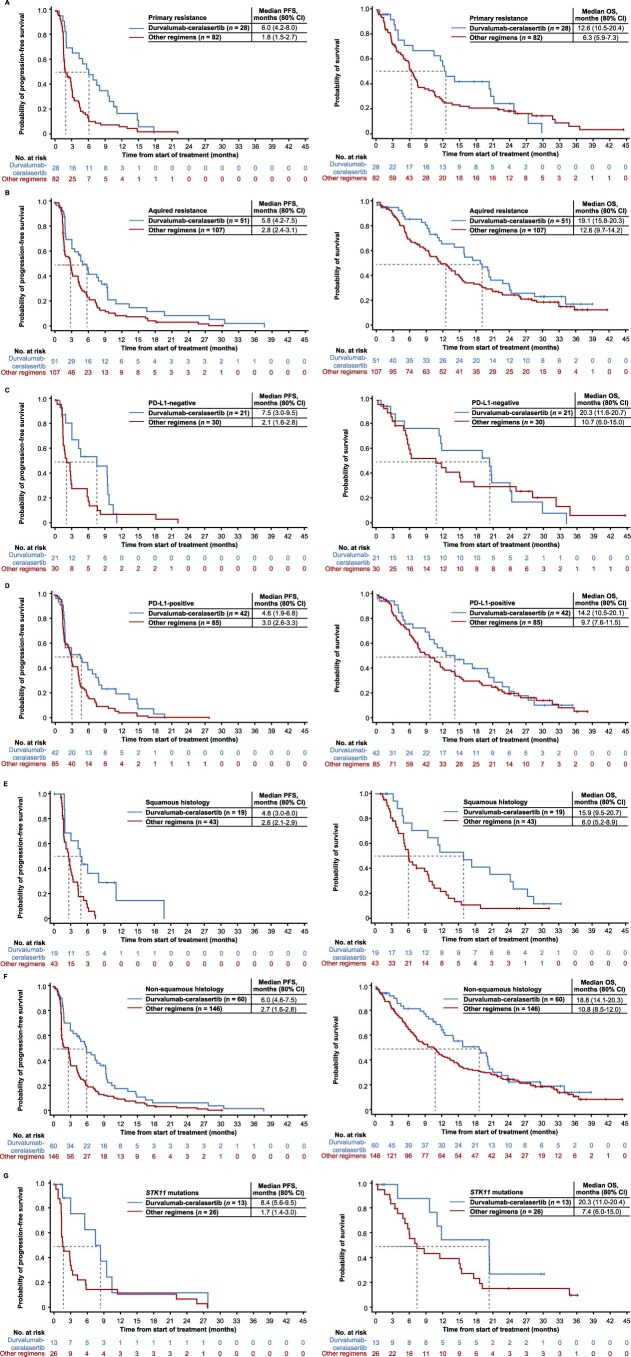
PFS and OS with durvalumab-ceralasertib and with durvalumab plus olaparib, danvatirsen or oleclumab in patient subgroups defined by presence or absence of adverse prognostic factors. Kaplan–Meier analyses of (left) PFS and (right) OS in patients with **a**, primary resistance, **b**, acquired resistance, **c**, PD-L1-negative tumours, **d**, PD-L1-positive tumours, **e**, squamous histology, **f**, non-squamous histology, and **g**, *STK11* mutations.

Given the encouraging observations with durvalumab–ceralasertib versus other regimens, we evaluated outcomes in subgroups defined by molecular and tumor-related features to determine whether specific factors were associated with benefit from, or resistance to, durvalumab–ceralasertib. Demographics and baseline disease characteristics in patients treated with durvalumab–ceralasertib or the other regimens by primary or acquired resistance and by histology are summarized in Supplementary Tables 11 and 12, respectively. In patients with primary resistance, median PFS (6.0 versus 1.8 months) and OS (12.6 versus 6.3 months) were longer with durvalumab–ceralasertib compared with the other regimens (Fig. 4 and Extended Data Fig. 3a), with nonoverlapping 80% CIs, with similar findings in patients with acquired resistance (median PFS 5.8 versus 2.8 months; median OS 19.1 versus 12.6 months) (Fig. 4 and Extended Data Fig. 3b). PFS and OS were also consistently longer in patients with PD-L1-negative (median PFS 7.5 versus 2.1 months; median OS 20.3 versus 10.7 months) or PD-L1-positive (median PFS 4.6 versus 3.0 months; median OS 14.2 versus 9.7 months) tumors (Fig. 4 and Extended Data Fig. 3c,d), and in patients with squamous (median PFS 4.8 versus 2.6 months; median OS 15.9 versus 6.0 months) or nonsquamous (median PFS 6.0 versus 2.7 months; median OS 18.8 versus 10.8 months) histology (Fig. 4 and Extended Data Fig. 3e,f). Similar findings were seen across all recalcitrant subgroups (Fig. 4), including those with liver or bone metastases, low TMB, and variant *STK11* (Extended Data Fig. 3g) and *KRAS*.

**Extended Data Fig. 4:**
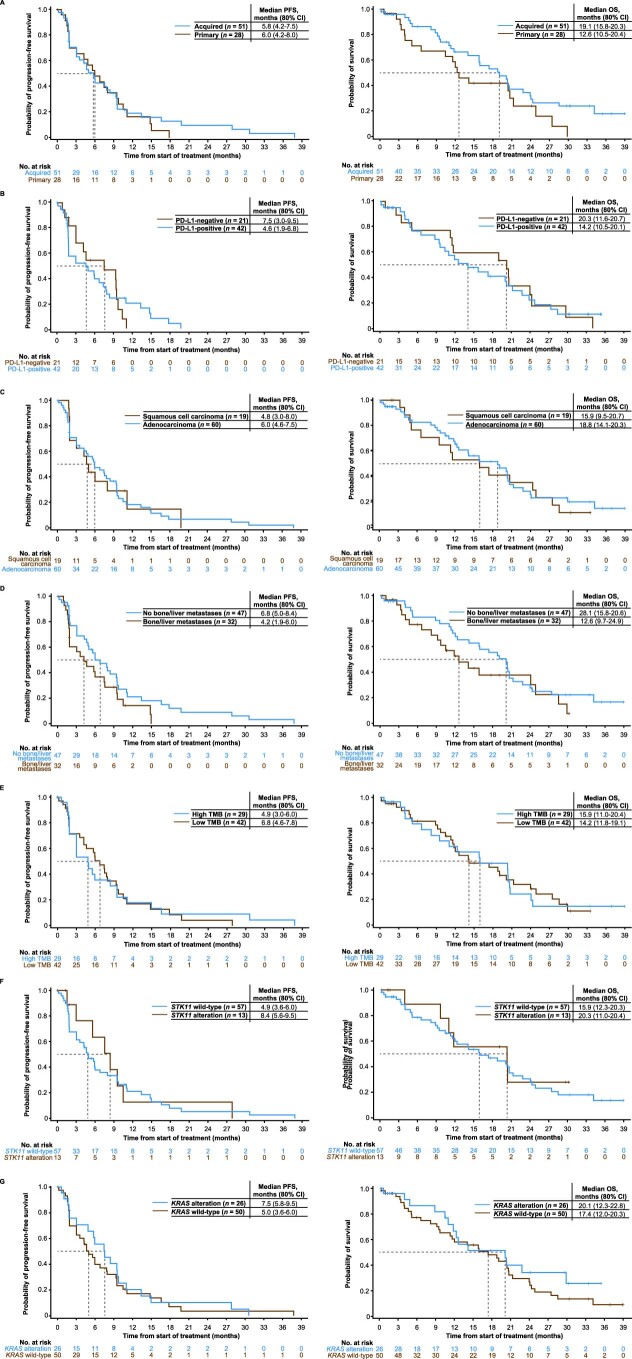
PFS and OS with durvalumab-ceralasertib in patient subgroups defined by presence or absence of adverse prognostic factors. Kaplan–Meier analyses of (left) PFS and (right) OS with durvalumab-ceralasertib in patients with **a**, primary vs acquired resistance, **b**, PD-L1-negative vs -positive status, and **c**, squamous cell carcinoma vs non-squamous histology, and in patients **d**, with or without bone/liver metastases, **e**, with high or low tumour mutational burden, and according to **f**, *STK11* status and **g**, *KRAS* status (variant or wild-type).

Kaplan–Meier analysis of PFS and OS with durvalumab–ceralasertib in subgroups defined by the presence or absence of the adverse prognostic factors described above (Extended Data Fig. 4) indicated that outcomes were similar in patients with primary or acquired resistance, PD-L1-negative or PD-L1-positive tumors, squamous or nonsquamous tumor histology, high or low TMB, and *STK11* or *KRAS* mutant or wild-type tumors. Presence of liver or bone metastases was associated with somewhat shorter PFS and OS, but benefit compared with the other durvalumab-based regimens was nevertheless evident in this setting (Fig. 4). Overall, these findings indicate that tumors with features commonly associated with immunotherapy resistance are nevertheless sensitive to durvalumab–ceralasertib.

We also explored differential gene alterations between patients with a PFS of ≥6 or <6 months on durvalumab–ceralasertib in the biomarker-matched (ATM altered) and biomarker-nonmatched (ATM wild-type) cohorts (Supplementary Fig. 1). A difference in alteration frequency was observed for *CDKN2A* alterations, which were more common in patients with PFS <6 months (*n* = 4/10, 40.0%) than in patients with PFS ≥6 months (*n* = 0/11) in the biomarker-matched cohort. The small sample sizes in these analyses limit interpretation of the findings.

### Ceralasertib immunomodulation enhances durvalumab effects

**Fig. 5:**
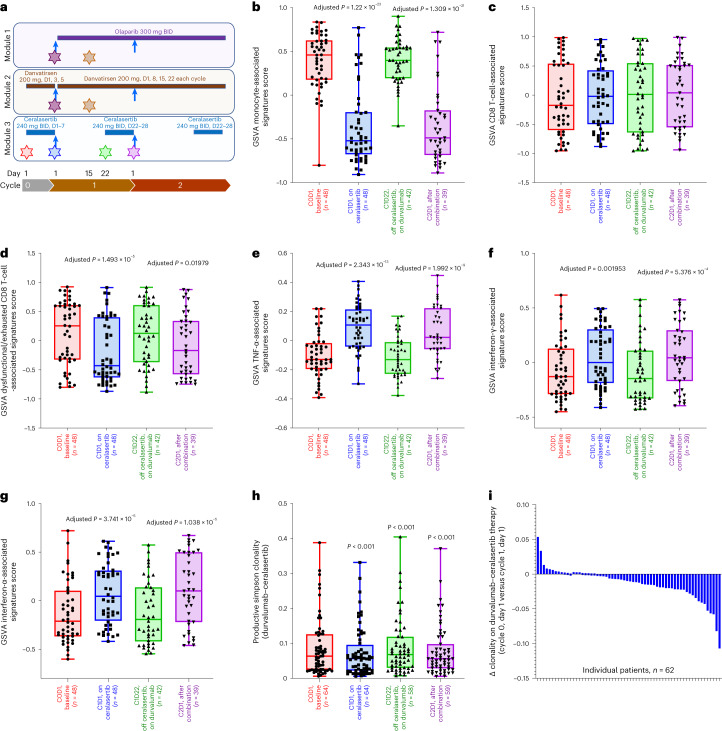

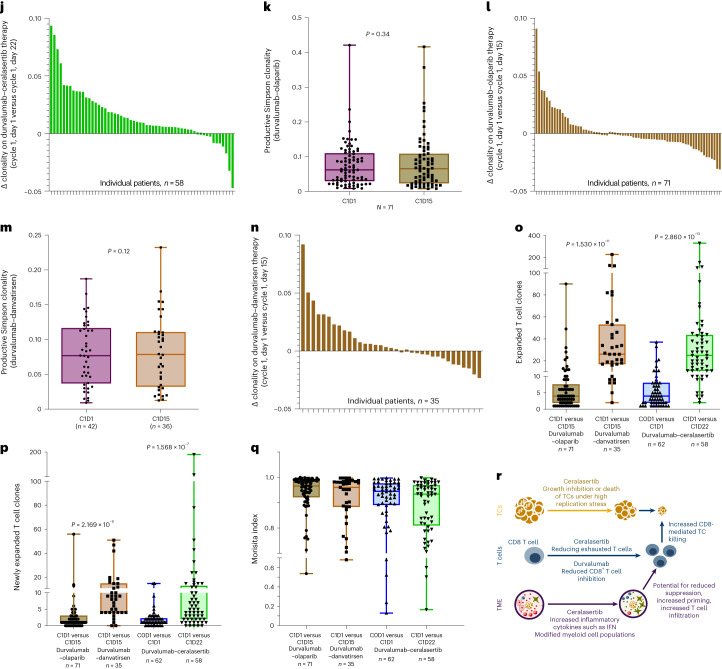
Ceralasertib induces systemic immunomodulation that enhances the immune-mediated effects of durvalumab in patients with NSCLC with progression on prior anti-PD-(L)1 treatment. **a**, Schema depicting treatment schedule for modules 1 (durvalumab–olaparib), 2 (durvalumab–danvatirsen) and 3 (durvalumab–ceralasertib), showing durvalumab administration (blue arrows), combination agent dosing (continuous colored bars), and pretreatment/on-treatment blood sample collection time points (stars). BID, twice daily. **b**–**g**, Effects of ceralasertib and durvalumab treatment on longitudinal blood-derived transcriptomes are depicted in the box plots showing differentially expressed monocyte-associated signatures (**b**), CD8 T-cell-associated signatures (**c**), dysfunctional/exhausted CD8 T-cell-associated signatures (**d**), TNF-α-associated signatures (**e**), interferon-γ-associated signatures (**f**) and interferon-α-associated signatures (**g**). *P* values are for comparisons with respective cycle 0, day (D) 1 time points. *P* values for visit dates represent linear mixed model effects, illustrating changes in on-treatment samples. **h**–**q**, Effects of ceralasertib, olaparib and danvatirsen with durvalumab on longitudinal blood-derived T cell repertoire: box plots showing productive Simpson T cell clonality with durvalumab–ceralasertib (**h**); waterfall plots showing changes in clonality on treatment by patient with durvalumab–ceralasertib on cycle 0, day 1 versus cycle 1, day 1 (**i**) and cycle 1, day 1 versus cycle 1, day 22 (**j**); box plots showing distributions of T cell clonality with durvalumab–olaparib (**k**), and waterfall plots showing changes in clonality on treatment by patient with durvalumab–olaparib on cycle 1, day 1 versus cycle 1, day 15 (**l**); box plots showing distributions of T cell clonality with durvalumab–danvatirsen (**m**), and waterfall plots showing changes in clonality on treatment per patient with durvalumab–danvatirsen on cycle 1, day 1 versus cycle 1, day 15 (**n**); box plots of expanded T cell clones (**o**), newly detected expanded T cell clones (**p**) and Morisita index for durvalumab plus olaparib, danvatirsen or ceralasertib at indicated visit dates (**q**). *P* values for visit dates represent two-sided Wilcoxon paired signed-rank tests between time points or two-sided Wilcoxon signed-rank tests between study modules, with correction for multiplicity of testing (Benjamini–Hochberg procedure), illustrating changes in on-treatment samples. **r**, Proposed immunomodulatory mechanism of action effects of ceralasertib with durvalumab in the periphery and tumor. For all box plots (**b**–**h**, **k**, **m** and **o**–**q**), box centerlines show medians, box limits show upper and lower quartiles, and whiskers show range.

Pretreatment and on-treatment blood biomarkers were evaluated in exploratory hypothesis-generating analyses using multi-omic approaches to assess potential underlying mechanisms of action with durvalumab–ceralasertib treatment (Fig. 5). Longitudinal matched blood samples were collected at baseline, after 7 days of ceralasertib therapy and after durvalumab treatment (Fig. 5a) to evaluate gene expression and T cell receptor (TCR) profile dynamics in 48 and 62 patients, respectively (patient demographics and disease characteristics in Supplementary Table 13). Gene expression analysis revealed dynamic, reversible changes following 7 days of ceralasertib and before the first dose of durvalumab. In addition, changes in innate and adaptive immunity-relevant signatures^51,52^ were seen, including decreases in monocyte lineage and CD8 T-cell-associated and dysfunctional/exhausted T-cell-associated signatures, and increases in the hallmark tumor necrosis factor (TNF)-α-associated, interferon-γ response-associated and interferon-α response-associated signatures (Fig. 5b–g).

Longitudinal TCR sequencing demonstrated cyclical changes including, most notably, a reduction in clonality after 7 days of ceralasertib (*P* < 0.0001), with a return to baseline after durvalumab treatment (Fig. 5h,i). An increase in peripheral TCR clonality was observed in most patients (*n* = 48/58) after the addition of durvalumab (Fig. 5h,j); similar changes in T cell clonality were not observed in samples from patients receiving durvalumab–olaparib (Fig. 5k,l) or durvalumab–danvatirsen (Fig. 5m,n). An increase in peripheral T cell clonality in patients receiving durvalumab–ceralasertib was followed by an increase in expanded clones (*P* < 0.0001) (Fig. 5o) and newly detected expanded T cell clones (*P* < 0.0001) (Fig. 5p) after the addition of durvalumab. The net result of changes in the circulating T cell repertoire after ceralasertib followed by durvalumab treatment was an overall increase in clonality in most patients, but without a significant difference in the composition of the most abundant T cell clones, as measured by the Morisita index (Fig. 5q). Overall, in peripheral blood, signatures indicating decreases in exhausted T cells and, conversely, increased interferon pathway activation were observed in the on-ceralasertib treatment period. Furthermore, we observed enhanced expansion and maintenance of abundant T cell clones indicative of antitumor response. These results suggest a complementary mechanism of action whereby ceralasertib induces systemic immunomodulation that may be indicative of an enhanced antitumor immune response in combination with durvalumab (Fig. 5r).

## Discussion

Critical challenges in the treatment of advanced NSCLC include identifying resistance mechanisms and finding new treatment options for use following failure of all standard-of-care therapies. For patients without targetable molecular alterations, this comprises treatment with platinum-doublet therapy and immunotherapy with an anti-PD-1/PD-L1 checkpoint inhibitor. Currently, there are few options offering clinical benefit for patients following failure of standard-of-care therapies^31^; therefore, one of the key aims of the HUDSON study is to identify regimens that can overcome inherent immune resistance in this setting. With its innovative, modular study design incorporating patient cohorts with specific biomarkers as well as biomarker-nonmatched cohorts with primary or acquired resistance, coupled with comprehensive characterization of key gene alterations via biological analysis of tumor material, HUDSON has enabled evaluation of multiple rational combinations and investigation of specific biomarkers associated with resistance.

Tumor-intrinsic factors and inflammation influence responses to ICB^13–16^. Higher TIS score is associated with anti-PD-(L)1 treatment benefit^49^ in the context of more substantial TME inflammation. We observed numerically higher TIS scores in subgroups with acquired resistance and adenocarcinoma histology compared with more ‘immune-cold’ subgroups with primary resistance and squamous cell histology. We found no differences in TIS score according to TMB or *STK11* status; this contrasts with published findings^49,53^ and may be associated with differences in biology between the post-ICB samples in HUDSON and ICB-naive samples in prior publications. Of note, liver and bone metastases are generally associated with immune-cold features; however, the biopsies we analyzed were not limited to these metastatic sites, which may explain the lack of association with inflammation in this subgroup of patients.

This first report from HUDSON demonstrates a notable efficacy signal with durvalumab–ceralasertib, with substantially higher response and disease control rates and substantially longer PFS and OS than with the other regimens, pooled. Furthermore, the median PFS and OS with durvalumab–ceralasertib of 5.8 and 17.4 months, respectively, are notable in the context of reports from prospective clinical trials in previously treated NSCLC of docetaxel as second- or third-line therapy following platinum-based chemotherapy and immunotherapy (median PFS and OS of 4.0 and 10.5 months in the CONTACT-01 study^54^; median OS of 8.3 months with docetaxel or pemetrexed (81%/19%) in the ATALANTE-1 study^55^; median PFS and OS of 4.5 and 11.3 months in primarily nonsquamous NSCLC in the CodeBreaK 200 study^56^) or following platinum-based chemotherapy (median PFS and OS ranges of 2.8–4.2 and 8.1–9.9 months, respectively^57–61^), in which findings were better than or similar to the data for the other HUDSON regimens, pooled. These outcomes indicate that durvalumab–ceralasertib warrants further investigation in this NSCLC patient population with a high unmet need for new treatment options^62^.

Durvalumab–ceralasertib had greater activity in the *ATM*-altered biomarker-matched cohort, with higher response rates and longer outcomes compared with biomarker-nonmatched cohorts. This observation is consistent with the study’s predefined hypothesis that *ATM*-altered patients would derive greater benefit from ATR inhibition with ceralasertib, associated with the known dependence of *ATM*-altered tumors on ATR signaling^63^. It should be noted that, while data were mature for the majority of patients of the reported modules, follow-up was limited for a small number of more recently enrolled patients receiving durvalumab–ceralasertib, some of whom were not evaluable for response and/or had not reached the 12-week time point at data cutoff. Nevertheless, findings from the *ATM* biomarker-matched cohort reflect the promising antitumor activity, notably in patients with alterations in the DDR pathway, seen in a study of durvalumab–ceralasertib in patients with advanced/metastatic melanoma following anti-PD-1 failure^64^. By contrast, the hypothesis of greater benefit with durvalumab plus the PARP inhibitor olaparib in patients with HRR mutations or *STK11* alterations did not appear to be borne out by our findings. Similarly, we did not see the hypothesized activity of durvalumab in combination with the STAT3 antisense oligonucleotide danvatirsen targeting the immunosuppressive TME or with the anti-CD73 monoclonal antibody oleclumab in patients with high CD73 expression.

Our subgroup analyses demonstrated broad activity with durvalumab–ceralasertib compared with the other regimens, including in patients with primary resistance, PD-L1-negative tumors, squamous histology, liver/bone metastases, low TMB, and *STK11* or *KRAS* variants; these exploratory analyses suggest that other subgroups such as patients with *RBM10* variants merit further investigation. These results suggest a potential sensitizing effect from the addition of ceralasertib to durvalumab in post-ICB NSCLC, and our data provide insights into the potential underlying mechanisms of action. Durvalumab–ceralasertib treatment induces systemic effects that associate with immune and inflammatory activities; thus, we hypothesize that these dynamic biomarkers could impact TME immunomodulation and thereby prime enhanced immune-mediated killing of TCs. Ongoing studies are currently investigating baseline and on-treatment tumor biopsies to further elucidate our mechanistic understanding of ceralasertib-mediated immune changes.

Durvalumab–ceralasertib demonstrated a generally acceptable safety profile and was broadly tolerable, with a low rate of treatment discontinuation due to TRAEs. Common TRAEs reflected those seen in previous studies^64,65^, with no new safety signals. Overall rates of TRAEs, grade ≥3 TRAEs and serious TRAEs were similar or numerically lower with durvalumab–ceralasertib compared with the other regimens, despite a >3-month longer mean duration of treatment. However, as with the efficacy data, these findings should be interpreted in the context of the limited follow-up in a number of patients receiving durvalumab–ceralasertib at data cutoff.

The heterogeneous nature of the NSCLC patient population in this setting highlights one of the benefits of platform or umbrella study designs such as in HUDSON, COAST^45^, NeoCOAST^66^ and Lung-MAP^67^. Modular designs provide the ability to evaluate multiple combinations within a specific treatment setting simultaneously. Additionally, in the context of investigating molecularly targeted treatment, such study designs can encompass patients with a range of different targetable aberrations, potentially resulting in an increased proportion of screened patients meeting specific cohort eligibility requirements, in contrast to single-arm phase 2 studies with a single set of eligibility criteria. Furthermore, central molecular screening of fresh biopsy samples, with molecular characterization of tumors, increased the chances of obtaining hypothesis-generating findings for both the biomarker-matched and biomarker-nonmatched primary and acquired resistance populations. However, the use of different NGS and immunohistochemistry assays for allocation of patients to the biomarker-matched or biomarker-nonmatched cohorts, and of archival or fresh biopsies, in HUDSON highlights the need for robust assessment using standardized biomarker testing with a short turnaround time.

HUDSON and platform studies in general also have limitations associated with their design. HUDSON was designed to allow the addition of new modules to adapt to developing understanding of anti-PD-(L)1 resistance mechanisms. Therefore, it is an open-label, nonrandomized study, and so it is not feasible to conduct formal controlled comparisons between treatment regimens, particularly with the limited cohort sizes. Additionally, because treating patients who had progressed on prior ICB with ICB alone was deemed to not be medically appropriate, it was not feasible to directly compare these combination regimens against durvalumab monotherapy, and so the specific benefit of adding each combination partner to durvalumab could not be determined. Comparison of outcomes between cohorts and regimens is further confounded by differences in patient demographics and baseline disease characteristics associated with the different subsets of patients treated within each cohort.

In conclusion, these findings have demonstrated an efficacy signal of interest with durvalumab–ceralasertib in advanced/metastatic NSCLC following prior failure of anti-PD-1/PD-L1 immunotherapy and platinum-doublet therapy. The regimen showed particular efficacy in patients with *ATM* alterations and in biomarker-nonmatched primary and acquired resistance cohorts across various subgroups recalcitrant to ICB. Our findings have thus resulted in initiation of the phase 3 LATIFY study (NCT05450692), which compares durvalumab–ceralasertib versus docetaxel for the treatment of patients with NSCLC whose disease progressed during or following prior anti-PD-1/PD-L1 therapy and platinum-based chemotherapy. Additionally, HUDSON is ongoing, with continued accrual to biomarker-matched and biomarker-nonmatched cohorts receiving durvalumab–ceralasertib or ceralasertib monotherapy and to cohorts investigating additional treatment combinations of durvalumab plus trastuzumab deruxtecan or cediranib.

## Methods

### Study design

HUDSON (NCT03334617) is an ongoing open-label, multicenter, umbrella phase 2 study of the anti-PD-L1 human monoclonal antibody durvalumab in combination with novel molecular-targeted anticancer agents in patients who have progressed on an anti-programmed death-1 (PD-1)/PD-L1-containing therapy and been exposed to a platinum-doublet regimen for locally advanced or metastatic NSCLC, either separately or in combination. HUDSON has a modular design, allowing initial assessment of the efficacy, safety and tolerability of multiple treatment combinations tailored by molecular alteration, with the goal of overcoming resistance to PD-(L)1 blockade.

The study is being performed in accordance with consensus ethical principles derived from international guidelines including the Declaration of Helsinki and Council for International Organizations of Medical Sciences International Ethical Guidelines, applicable International Council for Harmonisation Good Clinical Practice Guidelines, all applicable laws and regulations, and the AstraZeneca policy on Bioethics and Human Biological Samples. The study protocol, protocol amendments, informed consent form, investigator’s brochure and other relevant documents were reviewed and approved by the Institutional Review Board/Ethics Committee at each participating center: Austria—Wiener Gesundheitsverbund–Klinik Floridsdorf; Uniklinikum Salzburg; LBI for Lung Health, c/o Klinik Penzing, Wiener Gesundheitsverbund; Universitätsklinik Innsbruck. Canada—Princess Margaret Cancer Centre; William Osler Health System–Brampton Civic Hospital; Alberta Health Services; The Ottawa Hospital; Centre Hospitalier de l'Universite de Montreal. France—CHU Nantes Hôpital Laennec; Institut Gustave Roussy; Institut Bergonié; Hôpital Bichat–Claude-Bernard. Israel—Rabin Medical Center–Beilinson Hospital; Rambam Health Care Campus; Meir Hospital. South Korea—Samsung Medical Center; Seoul National University Hospital; Asan Medical Center. Spain—Hospital Universitario Ramón y Cajal; Hospital Universitario Virgen Macarena. United States—The University of Texas; Johns Hopkins University; UCLA; Washington University in St. Louis School of Medicine; St Joseph Heritage Healthcare; Dana Farber Mass General Brigham Cancer Care; City of Hope National Medical Center; Fox Chase Cancer Center; Sarah Cannon Research Institute at Tennessee Oncology; New York-Presbyterian; University of California San Diego; Sibley Memorial Hospital; Virginia Cancer Specialists; University of Pittsburgh School of Medicine. Protocol sections covering the modules reported in the manuscript are available as outlined in the data availability statement.

Patients are assigned to either a biomarker-matched group (group A) or a biomarker-nonmatched group (group B) based on tumor molecular profile at screening, as assessed by a consistent genomic testing protocol. Within the biomarker-matched group, patients with tumors with a mutation detected in a homologous recombination repair gene (HRRm) or with detectable aberrations in *LKB1* (liver kinase B1; also known as serine threonine kinase 11 (*STK11*)) were enrolled to separate cohorts to receive durvalumab plus the PARP inhibitor olaparib (module 1); patients with detectable aberrations in *ATM* (ataxia telangiectasia mutated) on next-generation sequencing (NGS; tissue-based FoundationOne CDx assay^68^ (Foundation Medicine) or circulating tumor DNA (ctDNA)-based GuardantHealth360 (Guardant Health) assay) or low ATM protein expression on immunohistochemistry (Ventana ATM (Y170) assay) received the ATR protein kinase inhibitor ceralasertib (AZD6738)^69^ (module 3); and patients with tumors expressing high levels of CD73 received durvalumab plus the anti-CD73 monoclonal antibody oleclumab (MEDI9447) (module 5). Other cohorts within the biomarker-matched group were opened or remain open for patient enrollment but are not described here as data are not sufficiently mature or planned initial accrual has not been completed. Within the biomarker-nonmatched group, patients without prespecified biomarkers are enrolled to cohorts according to whether they had primary or acquired resistance to their prior anti-PD-1/PD-L1 therapy, defined respectively as disease progression within ≤24 weeks or after >24 weeks from the start of treatment, while still on that treatment. At data cutoff for this report (modules 1, 3 and 5: 26 April 2022; module 2: 26 October 2020), data were available from biomarker-nonmatched cohorts treated with durvalumab plus olaparib (module 1), the signal transducer and activator of transcription 3 (STAT3) inhibitor danvatirsen (AZD9150) (module 2), ceralasertib (module 3) and oleclumab (module 5); other cohorts were opened or remain open. For each cohort within the biomarker-matched and biomarker-nonmatched groups, planned enrollment was 20 evaluable patients, with expansion to 40 evaluable patients determined on the basis of efficacy findings. Module 4, investigating durvalumab plus the dual mammalian target of rapamycin complex 1 and 2 (mTORC1/2) inhibitor vistusertib (AZD2014)^70^ in patients with RPTOR independent companion of MTOR complex 2 (RICTOR) mutations, was halted after one patient had been dosed due to discontinuation of drug development, and is not reported here.

The primary objective is to assess the objective response rate (ORR) for each treatment combination according to Response Evaluation Criteria for Solid Tumours (RECIST) version 1.1 (refs. ^71,72^). Secondary objectives include the assessment of disease control rate, PFS and OS, as well as the safety and tolerability of each treatment regimen. Exploratory objectives include investigations of cancer-relevant immune status, including biomarker analyses according to specific gene or protein expression profiles (for example, PD-L1), and the usage of subsequent anticancer therapy.

### Patients

To be eligible for enrollment, all patients had to be ≥18 years of age and to have histologically or cytologically confirmed metastatic or locally advanced and recurrent NSCLC that was progressing. Patients were required to be eligible for second- or later-line therapy and must have received an anti-PD-1/PD-L1-containing therapy and a platinum-doublet regimen for locally advanced or metastatic NSCLC either separately or in combination. Prior durvalumab was permitted. Patients must have had disease progression on a prior line of anti-PD-1/PD-L1 therapy. Patients needed to be suitable for a new tumor biopsy or to have had a biopsy post-progression on an anti-PD-1/PD-L1 containing therapy within approximately 3 months of screening, and required ≥1 lesion that could be accurately measured predose as ≥10 mm in the longest diameter (except for lymph nodes, for which the requirement was a short axis ≥15 mm) using computed tomography (CT) or magnetic resonance imaging and that was suitable for accurate repeated measurements. Additionally, patients were required to have a body weight of >30 kg, no cancer-associated cachexia, an Eastern Cooperative Oncology Group (ECOG) performance status of 0 to 1, a minimum life expectancy of 12 weeks, and a treatment-free interval of ≥3 weeks from any prior therapy before starting study treatment.

Patients were excluded if their tumors had targetable alterations in *EGFR* and/or *ALK* or were known to have targetable alterations in *ROS1*, *BRAF*, *MET* or *RET*. Patients must not have had toxicity that led to permanent discontinuation of prior anti-PD-1/PD-L1 immunotherapy, or any grade ≥3 immune-related adverse event (AE) or an immune-related neurologic or ocular AE of any grade while receiving prior immunotherapy. All AEs that occurred while receiving prior immunotherapy must have completely resolved or resolved to baseline before screening. Other exclusion criteria were the following: history of active primary immunodeficiency; active or prior documented autoimmune or inflammatory disorders (except vitiligo, alopecia, hypothyroidism that was stable on hormone replacement therapy, any chronic skin condition not requiring systemic therapy, or celiac disease controlled by diet alone); a history of allogenic organ transplantation; any uncontrolled intercurrent illness; spinal cord compression or symptomatic brain metastases; active infection; history of another primary malignancy (except malignancies treated with curative intent and with no known active disease ≥2 years before the first dose of study drug and of low potential risk for recurrence, adequately treated nonmelanoma skin cancer or lentigo maligna without evidence of disease, adequately treated carcinoma in situ without evidence of disease, and localized noninvasive primary under surveillance); small-cell lung cancer; presence of prespecified cardiac criteria; and inadequate bone marrow reserve or organ function.

Patients could not be receiving any concurrent chemotherapy, immunotherapy, biologic or hormonal therapy for cancer treatment, but use of hormones for non-cancer-related conditions and local treatment of isolated lesions, excluding target lesions, for palliative intent were acceptable. Current or prior use of immunosuppressive medication within 14 days before the first dose of durvalumab was not permitted (except for intranasal, inhaled, topical steroids or local steroid injections; systemic corticosteroids at doses not exceeding 10 mg per day of prednisone or its equivalent; steroids as premedication for hypersensitivity reactions). Patients were not allowed to have received a live attenuated vaccine within 30 days before the first dose of study drug, although authorized/approved coronavirus disease 2019 vaccines were permitted, with the recommendation to avoid administration for 72 h before the first dose of study drug.

All patients provided written informed consent. Prescreening consent was obtained for access to preexisting molecular information (if available), testing of an archival tumor sample (if available) and/or plasma ctDNA, and provision of a new tumor biopsy sample and blood sample for ctDNA testing. Following prescreening, and a valid molecular analysis result, patients were assigned to, and invited to consent to, a specific treatment cohort.

### Treatment and assessments

All patients received durvalumab 1,500 mg via intravenous infusion once every 4 weeks. Patients also received: olaparib orally at a dose of 300 mg twice daily (module 1 cohorts); danvatirsen intravenously at a dose of 200 mg every other day for a 1-week lead-in period before starting durvalumab and then weekly in combination with durvalumab (module 2 cohorts); ceralasertib orally at a dose of 240 mg twice daily on days 1–7 of a 1-week lead-in period before starting durvalumab and then on days 22–28 of each 4-week cycle in combination with durvalumab (module 3 cohorts); or oleclumab intravenously at a dose of 3,000 mg once every 2 weeks in cycles 1–2 and then once every 4 weeks from cycle 3 onwards (fixed dosing for patients with body weight >30 kg) (module 5 cohorts).

Tumors were assessed using contrast enhanced CT (or magnetic resonance imaging if CT was contraindicated) scans of the chest, abdomen and pelvis (including liver and adrenal glands) at baseline, every 6 weeks (±1 week) for the first 24 weeks from the start of combination therapy, and every 8 weeks (±1 week) thereafter until disease progression (confirmed with a subsequent scan). Patients who, in the opinion of the investigator, were still receiving clinical benefit from treatment, were permitted to continue treatment following confirmed radiological disease progression per RECIST v1.1 (refs. ^71,72^). Scheduled radiological scans were continued for these patients while they were still receiving study treatment. Objective tumor responses were assessed and categorized programmatically per RECIST v1.1 (refs. ^71,72^). Survival status was recorded every 3 months (±1 week) after a safety follow-up visit (90 days after study drug discontinuation) and until the earlier of: 12 months after the last patient had discontinued treatment in the last cohort within a module; or 75% of patients had died in all cohorts within a module. Safety was assessed and AEs recorded throughout the treatment period and the safety follow-up (90 days after the discontinuation of all study drugs or until initiation of another therapy, unless the investigator assesses that the event occurring within 90 days after last dose of study treatment but after the initiation of another therapy is related to the study treatment). Severity of AEs was assessed per the National Cancer Institute’s Common Terminology Criteria for Adverse Events version 4.03. Causal relationship between study drug and each AE was assessed by the investigators, by answering ‘yes’ or ‘no’ to the question ‘Do you consider that there is a reasonable possibility that the event may have been caused by the study drug?’.

### Biomarker analyses

A collection of tumor samples, including both resections (archival) and post-anti-PD-1/PD-L1 progression biopsies (new), was assembled from patients during screening. Each sample was sequenced using the Foundation Medicine NGS FoundationOneCDx assay^68^. The assay detects mutations and copy number variations in 324 cancer-related genes and selected rearrangements. The assay also provides information on microsatellite instability and TMB. Detailed information on the assay and the variant calling pipeline are available at ref. ^73^. TMB is reported as low (<10 mutations Mb^−1^) or high (≥10 mutations Mb^−1^). Mutation profiles included co-occurring and mutually exclusive gene alterations. For each gene, substitutions, short insertions and deletions, rearrangements and copy number changes of known or likely functional relevance detected using the assay were included. Additionally, tumor PD-L1 expression was assessed locally using PD-L1 immunohistochemistry assays. PD-L1 expression was evaluated in TCs and considered positive when expressed in ≥1% of neoplastic cells.

### RNA extraction and whole transcriptome library preparation

Bulk-RNA sequencing was conducted on suitable fresh biopsies acquired from a subset of patients with evaluable prescreening tumor biopsies, before patient allocation to a HUDSON treatment module. Fresh frozen core needle biopsy samples were divided (where possible) while frozen on dry ice, with one portion consumed for total RNA extraction. Total RNA was extracted using Qiagen RNeasy Mini kit (Qiagen, cat. no. 74104) with on-column DNase digestion (cat. no. 79254) and eluted in 30 ml nuclease-free water. RNA concentration, RNA integrity number and %DV200 (percentage of RNA fragments >200 nucleotides) were determined using Agilent RNA ScreenTapes or Agilent High Sensitivity RNA ScreenTapes via Agilent TapeStation. Total RNA was arrayed on a 96-well polymerase chain reaction plate, and whole transcriptome libraries were generated using KAPA RNA Hyper Prep Kit with RiboErase (HMR) Globin (Roche cat. no. KK8563), in accordance with the manufacturer’s protocol. Library concentrations were determined using Agilent D1000 ScreenTapes on Agilent TapeStation. Whole transcriptome sequencing libraries were sequenced with the Illumina NovaSeq 6000 kit v1.5 300 cycles (150 × 2).

Transcript-per-million (TPM)-normalized bulk-RNA sequencing data were available for 49 prescreening samples from 49 unique patients. The TPM matrix was used as input for single-sample gene set enrichment analysis (ssGSEA) as implemented in GSVA R package version 1.42.0 to compute enrichment scores (ES) for the 18-gene TIS^47^. ssGSEA ES were compared between patients with immunotherapy-refractory/relapsed NSCLC, including recalcitrant subgroups, using the two-sided Wilcoxon rank-sum test. Specifically, TIS ssGSEA ES was compared between patients with primary (progression within 24 weeks) or acquired (progression after 24 weeks) resistance to prior ICB therapy, patients with PD-L1-positive (≥1% positive TCs based on immunohistochemical staining) or PD-L1-negative (<1% TCs) tumors, patients with or without bone and/or liver metastases, and patients with adenocarcinoma or squamous cell tumors. In addition, comprehensive genomic profiling using targeted next-generation sequencing was performed on archived or baseline tissue samples using the FoundationOne CDx assay^68^. Detailed information on the assay and the variant calling pipeline is available at ref. ^73^. Based on the mutational status, TIS ssGSEA ES were compared between patients with S*TK11*-altered or *STK11*-wild-type tumors, and patients with TMB-low (<10 mutations Mb^−1^) or TMB-high (≥10 mutations Mb^−1^) tumors. The TPM matrix was *z*-scored gene-wise to display the expression level of the 18 genes composing the TIS as heat maps. It is important to note that analyses were not restricted to specific biopsy sites because of the limited numbers of prescreening biopsies assessed using bulk-RNA sequencing.

### RNA extraction and NanoString gene expression

Total RNA was extracted from pre- and posttreatment blood samples using the PAXgene Blood RNA Kit (PreAnalytiX GmbH) per the manufacturer′s recommended protocol. RNA concentration and quality were assessed on the TapeStation 2200 System (Agilent). nCounter gene expression assays (NanoString Technologies) were performed on patients’ blood RNA samples using the 770-gene human PanCancer Immune Profiling panel^74^ (NanoString Technologies) and a custom 30-gene spike-in panel, per NanoString′s recommended protocol. The custom spike-in panel target genes included *APOBEC3B*, *B2M*, *CALB2*, *CD69*, *CGAS*, *CMPK2*, *DHX58*, *EGFR*, *GBP1*, *GBP4*, *GBP5*, *GDF15*, *HAP1*, *HOPX*, *IFNG*, *IL12A*, *KEAP1*, *NKG7*, *OASL*, *PF4*, *PROM1*, *RGS16*, *RSAD2*, *SAP30*, *SLAMF8*, *STING1*, *STK11*, *TREX1*, *TSLP* and *VTCN1*. Raw count data were preprocessed using background subtraction and housekeeping gene normalization functions in nSolver 4.0 (NanoString Technologies). The data that passed quality control using nSolver’s default quality control parameters were then analyzed using a linear mixed effects model.

### TCR sequencing and analysis

Deep sequencing of the CDR3 regions of TCR-β genes was performed by Adaptive Biotechnologies using the immunoSEQ assay on genomic DNA purified with the Qiagen DNeasy Blood extraction kit from total peripheral blood mononuclear cells obtained at each study visit. Only productive (in-frame) TCR sequences were considered in the analyses^75,76^. To identify expanded and contracted T cell clones between samples from an individual patient at different visits, we used the Fisher exact test to compute a *P* value for each TCR clonotype across the two samples, against the null hypothesis that the population abundance of the clone is identical in the two samples. We corrected for multiple testing to control the false discovery rate using the Benjamini–Hochberg procedure and employed a significance threshold of 0.01 on adjusted *P* values. Expanded clones not observed at baseline and detected above a threshold of five reads were classified as newly detected expanded clones. We calculated Simpson’s clonality (as 1 − Pielou’s evenness), which normalizes sampling depth to compare TCR repertoires between samples. To measure the stability of the clonotypes in the circulating repertoire across visits, we used the Morisita–Horn similarity index, which accounts for the number of common clonotypes and the distribution of clonotype sizes and is most sensitive to the clone sizes of the dominant clonotypes^75,76^. Wilcoxon paired signed-rank tests were used to compare groups of matched samples.

### Statistical analysis

Initial sample size for each cohort was to be 20 evaluable patients, with possible expansion based on observation of an efficacy signal per the primary endpoint of ORR. Evaluations were to be carried out after the ~20th evaluable patient in a cohort, or the final patient dosed in a cohort if enrollment ended early, had had the opportunity for two on-treatment response assessments per RECIST or had discontinued or withdrawn from treatment. A statistical framework was built around a prespecified ORR to inform the Sponsor if an efficacy signal had been observed and a recommendation to expand should be made. Decisions regarding stopping recruitment in specific cohorts were at the discretion of the Sponsor and were based on emerging efficacy, safety and tolerability data.

PFS was defined as the time from start of treatment until the date of objective disease progression according to RECIST 1.1 or death (by any cause in the absence of progression) regardless of whether the patient withdrew from therapy or received another anticancer therapy before progression. Patients who had not progressed or died at the time of analysis were censored at the time of the latest date of assessment from their last evaluable RECIST 1.1 assessment. However, if a patient progressed or died after two or more missed visits, the patient was censored at the time of the latest evaluable RECIST 1.1 assessment before the two missed visits. OS was defined as the time from the start of treatment until death due to any cause. Any patient not known to have died at the time of analysis was censored on the basis of the last recorded date on which the patient was known to be alive. PFS and OS were evaluated using Kaplan–Meier methodology. Analyses of clinical data were conducted using SAS v9.3.

Data cutoff for modules 1, 3 and 5 was 26 April 2022. Due to the nature of this platform study, which enrolled different cohorts at different time periods, data cutoff for module 2 was 26 October 2020.

### Reporting summary

Further information on research design is available in the Nature Portfolio Reporting Summary linked to this article.

## Online content

Any methods, additional references, Nature Portfolio reporting summaries, source data, extended data, supplementary information, acknowledgements, peer review information; details of author contributions and competing interests; and statements of data and code availability are available at 10.1038/s41591-024-02808-y.

### Supplementary information

Supplementary Information Supplementary Tables 1–13 and Fig. 1.

Reporting Summary

## Figures and Tables

**Extended Data Table 1:**
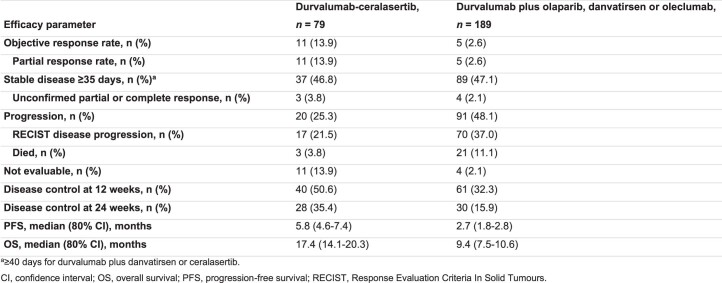
Treatment efficacy by combination regimen: durvalumab-ceralasertib versus durvalumab plus olaparib, danvatirsen or oleclumab

Analyses by individual cohort are provided in Extended Data Table 2 and Supplementary Tables 3–5.

**Extended Data Table 2:**
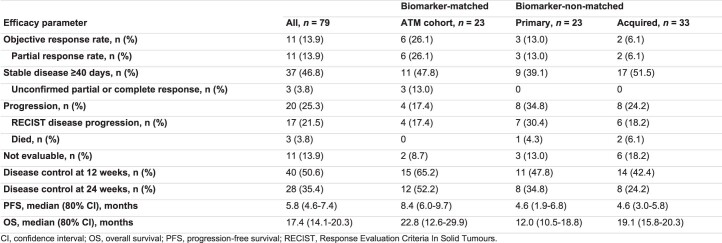
Treatment efficacy with durvalumab-ceralasertib, by cohort

**Extended Data Table 3:**
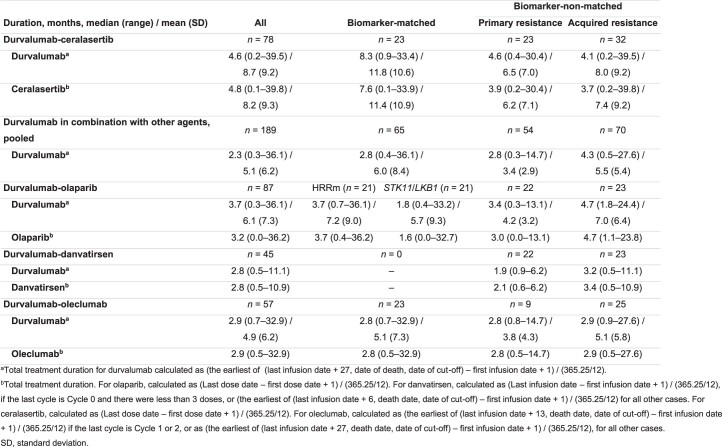
Duration of treatment by cohort

**Extended Data Table 4:**
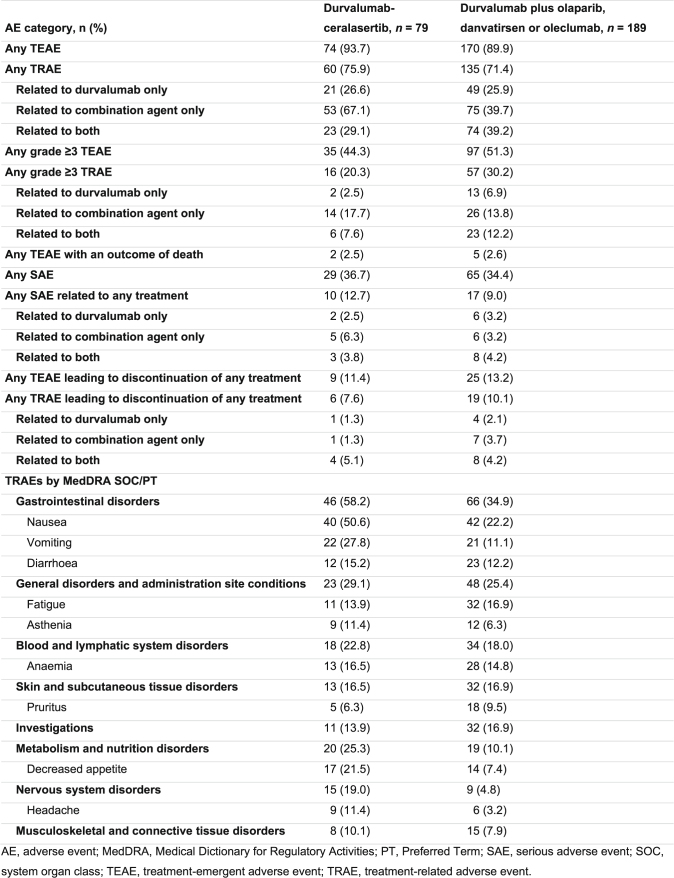
Safety profile and incidence of treatment-related adverse events according to MedDRA SOC and PT (≥10% in either group) by combination regimen: durvalumab-ceralasertib versus durvalumab plus olaparib, danvatirsen or oleclumab

Analyses by individual cohort are provided in Supplementary Tables 6–9.

**Extended Data Table 5:**
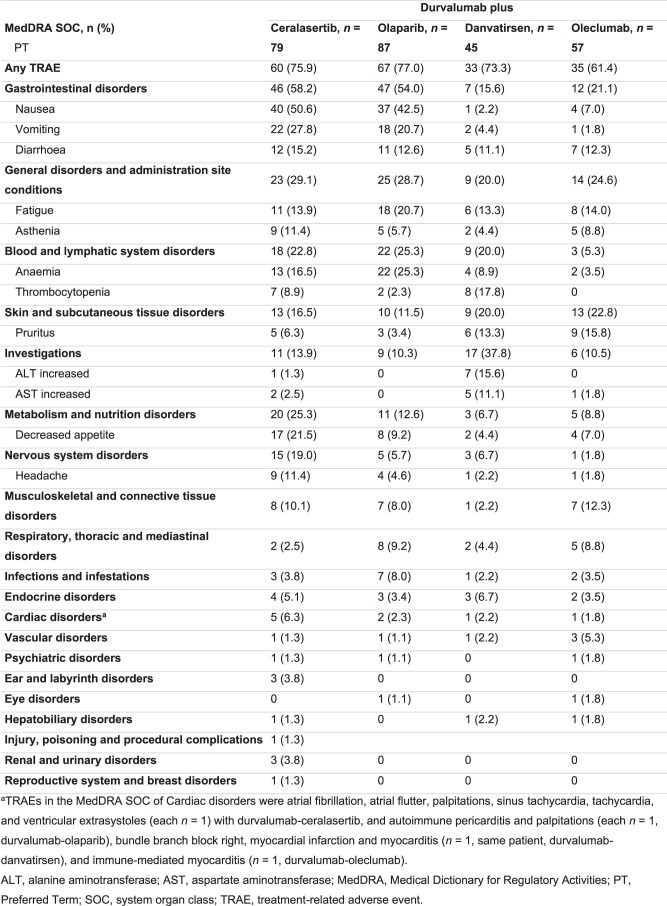
Incidence of treatment-related adverse events according to system organ class (all) and preferred term (≥10% in any treatment cohort), by combination regimen

**Extended Data Table 6:**
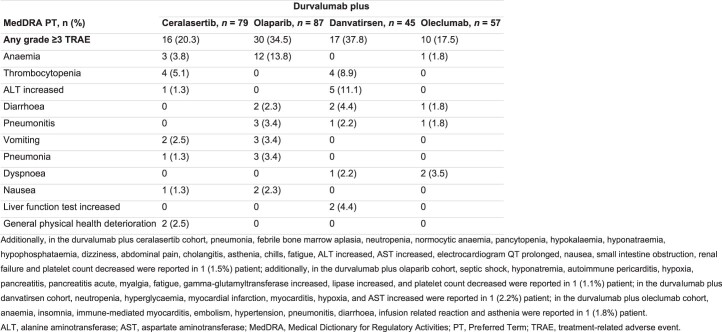
Incidence of grade ≥3 treatment-related adverse events according to preferred term (≥2 patients in any treatment cohort), by combination regimen

## Data Availability

The relevant sections of the study protocol, plus data underlying the findings described in this paper, may be obtained in accordance with AstraZeneca’s data sharing policy described at https://astrazenecagrouptrials.pharmacm.com/ST/Submission/Disclosure. Data for studies directly listed on Vivli can be requested through Vivli at www.vivli.org. Data for studies not listed on Vivli could be requested through Vivli at https://vivli.org/members/enquiries-about-studies-not-listed-on-the-vivli-platform/. AstraZeneca’s Vivli member page is also available outlining further details (https://vivli.org/ourmember/astrazeneca/).
