# Supplementary material for: Biomarker-directed targeted therapy plus durvalumab in advanced non-small-cell lung cancer: a phase 2 umbrella trial
Source: Nat Med. 2024 Feb 13;30(3):716–29. doi: 10.1038/s41591-024-02808-y (PMC10957481; doi:10.1038/s41591-024-02808-y)
Supplement: Supplementary file 1 — Supplementary Tables 1–13 and Fig. 1. [file 41591_2024_2808_MOESM1_ESM.pdf]

nature medicine

Article https://doi.org/10.1038/s41591-024-02808-y

Biomarker-directed targeted therapy plus durvalumab in advanced non-small-cell lung cancer: a phase 2 umbrella trial

In the format provided by the authors and unedited

Biomarker-directed targeted therapy plus durvalumab in advanced non-small-cell lung cancer: a phase 2 umbrella trial

Authors:

Benjamin Besse, Elvire Pons-Tostivint, Keunchil Park, Sylvia Hartl, Patrick M. Forde, Maximilian J. Hochmair, Mark M. Awad, Michael Thomas, Glenwood Goss, Paul Wheatley-Price, Frances A. Shepherd, Marie Florescu, Parneet Cheema, Quincy S.C. Chu, Sang-We Kim[14], Daniel Morgensztern[15], Melissa L. Johnson[16], Sophie Cousin[17], Dong-Wan Kim[18], Mor T. Moskovitz[19], David Vicente Baz[20], Boaz Aronson[21], Rosalind Hobson[22], Helen J. Ambrose[23], Sajan Khosla[24], Avinash Reddy[25], Deanna L. Russell[26], Mohamed Reda Keddar[27], James P. Conway[28], J. Carl Barrett[29], Emma Dean[30], Rakesh Kumar[31], Marlene Dressman[31], Philip J. Jewsbury[30], Sonia Iyer[29], Simon T. Barry[30], Jan Cosaert[30] and John V. Heymach[32]

Supplementary Information

- Supplementary Tables 1–13
- Supplementary Fig. 1

Supplementary Tables

Supplementary Table 1 | Prior immunotherapy, best response on prior immunotherapy, and time from prior immunotherapy to starting durvalumab plus ceralasertib or durvalumab plus olaparib, danvatirsen or oleclumab

Responders	Prior anti-PD-(L)1/anti-CTLA4 immunotherapy regimen(s)[a]	Best response on prior immunotherapy regimen(s)	Duration on prior immunotherapy regimen(s), months	Time from end of prior anti-PD(L)1 immunotherapy to enrollment in HUDSON, months	Other regimens received prior to HUDSON[a]	Resistance classification on HUDSON
**Patients with PR on durvalumab-ceralasertib**						
**1**	Anti-PD-L1	PR	27.2	4.0	Platinum doublet + 1 other	Acquired
**2**	Anti-PD-L1	PR	5.2	2.1	Platinum doublet + 1 other	Acquired
**3**	Anti-PD-L1	SD	17.3	14.0	Platinum doublet + 1 other	Acquired
**4**	Anti-PD-L1	NE	NC	17.2	Platinum doublet + 3 others	Primary
**5**	Anti-PD-L1	SD	31.3	2.3	Platinum doublet + 2 others	Acquired
**6**	Anti-PD-L1	SD	2.6	2.1	Platinum doublet + 1 other	Primary
**7**	Anti-PD-L1	SD	32.2	3.2	Platinum doublet + 2 others	Acquired
**8**	Anti-PD-L1	SD	2.9	16.6	Platinum doublet + 3 others	Primary
**9**	Anti-PD-L1	PD	1.5	17.8	Platinum doublet + 2 others	Primary
**10**	Anti-PD-L1	SD	8.5	2.1	Platinum doublet + 1 other	Primary
**11**	Anti-PD-L1	PD	1.4	14.7	Platinum doublet + 2 others	Acquired[b]
**Patients with PR on durvalumab-olaparib**						
**1**	ICI – Anti-PD-L1	PR	40.1	1.5	Platinum doublet + 3 others	Acquired
**2**	ICI – Anti-PD-L1	SD	5.4	0.9	Platinum doublet + 1 other	Primary
**3**	ICI – Anti-PD-L1	PR	24.0	1.1	Platinum doublet + 1 other	Acquired
**4**	ICI – Anti-PD-L1	SD	8.9	3.4	Platinum doublet + 1 other	Acquired
**Patient with PR on durvalumab-oleclumab**						
**1**	ICI – Anti-PD-L1	PR	8.7	13.4	Platinum doublet + 3 others	Acquired

[a]Detailed regimen information not provided for individual patients to minimise potential for individual patient identification.

[b]Patient had 6 weeks of ICI therapy and relapsed on day 425, thus meeting the criteria for having acquired resistance despite the short treatment duration.

ICI, immune checkpoint inhibitor; NC, not calculated due to incomplete start date; NE, non-evaluable; PD, progressive disease; PR, partial response; SD, stable disease.

Supplementary Table 2 | Baseline characteristics in patients receiving durvalumab plus ceralasertib or durvalumab plus olaparib, danvatirsen or oleclumab, in the biomarker-matched and biomarker-non-matched groups.

Characteristic	Biomarker-matched		Biomarker-non-matched	
	Durvalumab-ceralasertib (ATM cohort), *n* = 23	Durvalumab plus olaparib, danvatirsen or oleclumab, *n* = 65	Durvalumab-ceralasertib, *n* = 56	Durvalumab plus olaparib, danvatirsen or oleclumab, *n* = 124
Age, median (range), years	63.0 (47-76)	61.0 (35-85)	64.0 (42-80)	65.5 (39-82)
Age <65 years, n (%)	15 (65.2)	43 (66.2)	30 (53.6)	59 (47.6)
Age ≥65 years, n (%)	8 (34.8)	22 (33.8)	26 (46.4)	65 (52.4)
Male sex, n (%)[a]	11 (47.8)	37 (56.9)	41 (73.2)	66 (53.2)
Race, n (%)[b]	*n* = 23	*n* = 64	*n* = 54	*n* = 123
White	17 (73.9)	49 (76.6)	30 (55.6)	75 (61.0)
Asian	0	5 (7.8)	9 (16.7)	33 (26.8)
Black or African American	0	0	3 (5.6)	4 (3.3)
Native Hawaiian or Other Pacific Islander	0	1 (1.6)	0	0
Other[c]	6 (26.1)	9 (14.1)	12 (22.2)	11 (8.9)
ECOG PS, n (%)[b]	*n* = 23	*n* = 65	*n* = 56	*n* = 123
0	10 (43.5)	17 (26.2)	18 (32.1)	47 (38.2)
1	13 (56.5)	47 (72.3)	38 (67.9)	76 (61.8)
2[d]	0	1 (1.5)	0	0
Histology, n (%)				
Adenocarcinoma	21 (91.3)	50 (76.9)	34 (60.7)	81 (65.3)
Squamous cell carcinoma	0	7 (10.8)	19 (33.9)	36 (29.0)
Large-cell carcinoma (NOS)	2 (8.7)	3 (4.6)	0	3 (2.4)
Other	0	5 (7.7)	3 (5.4)	4 (3.2)
Time from diagnosis, n (%)[b]	*n* = 23	*n* = 63	*n* = 56	*n* = 121
≤12 months	3 (13.0)	17 (27.0)	10 (17.9)	27 (22.3)
>12 months	20 (87.0)	46 (73.0)	46 (82.1)	94 (77.7)
Disease classification, n (%)[b]	*n* = 23	*n* = 65	*n* = 56	*n* = 123

Characteristic	Biomarker-matched		Biomarker-non-matched	
	Durvalumab-ceralasertib (ATM cohort), *n* = 23	Durvalumab plus olaparib, danvatirsen or oleclumab, *n* = 65	Durvalumab-ceralasertib, *n* = 56	Durvalumab plus olaparib, danvatirsen or oleclumab, *n* = 124
Metastatic	23 (100)	64 (98.5)	54 (96.4)	120 (97.6)
Locally advanced	0	1 (1.5)	2 (3.6)	3 (2.4)
Metastatic sites, n (%)				
≤2	9 (39.1)	35 (53.8)	22 (39.3)	77 (62.1)
≥3	14 (60.9)	30 (46.2)	34 (60.7)	47 (37.9)
Metastatic site location, n (%)				
Bone and locomotor	10 (43.5)	24 (36.9)	14 (25.0)	31 (25.0)
Adrenal gland	5 (21.7)	13 (20.0)	10 (17.9)	24 (19.4)
Brain/CNS and/or other CNS	7 (30.4)	15 (23.1)	5 (8.9)	21 (16.9)
Liver and/or hepatic (including gall bladder)	6 (26.1)	16 (24.6)	8 (14.3)	19 (15.3)
PD-L1 status, n (%)				
Positive (TC ≥1%)	12 (52.2)	23 (35.4)	30 (53.6)	62 (50.0)
1–49%	7 (30.4)	12 (18.5)	18 (32.1)	34 (27.4)
≥50%	5 (21.7)	11 (16.9)	12 (21.4)	28 (22.6)
Negative (TC <1%)	5 (21.7)	13 (20.0)	16 (28.6)	17 (13.7)
Unknown	6 (26.1)	29 (44.6)	10 (17.9)	44 (35.5)
Missing	0	0	0	1 (0.8)
Prior regimens, n (%)				
1	4 (17.4)	10 (15.4)	11 (19.6)	12 (9.7)
2	9 (39.1)	28 (43.1)	18 (32.1)	57 (46.0)
3	4 (17.4)	14 (21.5)	18 (32.1)	33 (26.6)
≥4	6 (26.1)	13 (20.0)	9 (16.1)	22 (17.7)
Prior immunotherapies, n (%)				
1	22 (95.7)	65 (100)	55 (98.2)	123 (99.2)
2	1 (4.3)	0	1 (1.8)	1 (0.8)

Characteristic	Biomarker-matched		Biomarker-non-matched	
	**Durvalumab-ceralasertib (ATM cohort), *n* = 23**	**Durvalumab plus olaparib, danvatirsen or oleclumab, *n* = 65**	**Durvalumab-ceralasertib, *n* = 56**	**Durvalumab plus olaparib, danvatirsen or oleclumab, *n* = 124**
**Prior anti-PD-(L)1 immunotherapy, n (%)**				
**Nivolumab**	9 (39.1)	30 (46.2)	27 (48.2)	63 (50.8)
**Pembrolizumab**	12 (52.2)	19 (29.2)	21 (37.5)	33 (26.6)
**Atezolizumab**	2 (8.7)	9 (13.8)	5 (8.9)	17 (13.7)
**Durvalumab**	0	7 (10.8)	1 (1.8)	10 (8.1)
**Cemiplimab**	0	0	0	1 (0.8)
**Prior anti-CTLA4 immunotherapy, n (%)**				
**Tremelimumab**	0	3 (4.6)	1 (1.8)	2 (1.6)
**Ipilimumab**	0	2 (3.1)	0	0
**Best response on prior immunotherapy, n (%)**				
**Complete response**	0	0	0	2 (1.6)
**Partial response**	7 (30.4)	14 (21.5)	10 (17.9)	40 (32.3)
**Stable disease**	11 (47.8)	27 (41.5)	29 (51.8)	37 (29.8)
**Stable disease, biomarker-matched patients**	11 (47.8)	27 (41.5)	0	0
**Stable disease, biomarker-non-matched patients**	0	0	29 (51.8)	37 (29.8)
**Progressive disease**	3 (13.0)	23 (35.4)	13 (23.2)	34 (27.4)
**Non-evaluable**	1 (4.3)	1 (1.5)	2 (3.6)	9 (7.3)
**Not applicable**	1 (4.3)	0	0	1 (0.8)
**Time from prior immunotherapy**	*n* = 23	*n* = 65	*n* = 54	*n* = 123
**Median, months (range)**	3.3 (0.8–20.5)	3.4 (0.9–50.1)	4.0 (0.7–31.4)	3.2 (0.7–30.3)
**Prior immunotherapy and resistance classification**				
**Primary resistance, n (%)**	7 (30.4)	27 (41.5)	22 (39.3)	53 (42.7)
**Prior monotherapy**	4 (17.4)	20 (30.8)	18 (32.1)	43 (34.7)
**Prior combination**	1 (4.3)	6 (9.2)	4 (7.1)	10 (8.1)

Characteristic	Biomarker-matched		Biomarker-non-matched	
	Durvalumab-ceralasertib (ATM cohort), *n* = 23	Durvalumab plus olaparib, danvatirsen or oleclumab, *n* = 65	Durvalumab-ceralasertib, *n* = 56	Durvalumab plus olaparib, danvatirsen or oleclumab, *n* = 124
Both monotherapy and combination	2 (8.7)	1 (1.5)	0	0
Acquired resistance, n (%)	16 (69.6)	38 (58.5)	32 (57.1)	70 (56.5)
Prior monotherapy	13 (56.5)	28 (43.1)	23 (41.1)	57 (46.0)
Prior combination	1 (4.3)	9 (13.8)	6 (10.7)	9 (7.3)
Both monotherapy and combination	2 (8.7)	1 (1.5)	3 (5.4)	4 (3.2)
Not available, n (%)	0	0	2 (3.6)	1 (0.8)
Prior platinum-based therapies, n (%)				
1	18 (78.3)	56 (86.2)	44 (78.6)	98 (79.0)
2	3 (13.0)	7 (10.8)	12 (21.4)	21 (16.9)
≥3	2 (8.7)	2 (3.1)	0	5 (4.0)
Smoking status, n (%)				
Never	1 (4.3)	10 (15.4)	8 (14.3)	18 (14.5)
Current	7 (30.4)	10 (15.4)	11 (19.6)	13 (10.5)
Former	15 (65.2)	45 (69.2)	37 (66.1)	93 (75.0)

[a]Sex as recorded by investigator in the case report form. [b]For parameters for which data are missing, the numbers of patients with data are indicated for each regimen and are used as the denominators for calculating percentages. [c]Recorded as ‘Other’ in the case report form. [d]Patient did not meet the eligibility criterion for ECOG PS of 0–1. [e]Sample was recorded by the investigator/site as PD-L1-positive, i.e. TC ≥1%, but absolute value of %TC positivity not provided to enable further sub-classification.

CNS, central nervous system; ECOG PS, Eastern Cooperative Oncology Group performance status; NOS, not otherwise specified; PD-L1, programmed cell death ligand 1.

Supplementary Table 3 | Treatment efficacy with durvalumab-olaparib, by cohort.

Efficacy parameter	All, *n* = 87	Biomarker-matched: HRRm cohort, *n* = 21	Biomarker-matched: *STK11/LKB1* cohort, *n* = 21	Biomarker-non-matched: Primary, *n* = 22	Biomarker-non-matched: Acquired, *n* = 23
**Objective response rate, n (%)**	4 (4.6)	2 (9.5)	1 (4.8)	0	1 (4.3)
**Partial response rate, n (%)**	4 (4.6)	2 (9.5)	1 (4.8)	0	1 (4.3)
**Stable disease ≥35 days, n (%)**	40 (46.0)	9 (42.9)	5 (23.8)	13 (59.1)	13 (56.5)
**Unconfirmed partial or complete response, n (%)**	1 (1.1)	1 (4.8)	0	0	0
**Progression, n (%)**	42 (48.3)	10 (47.6)	15 (71.4)	8 (36.4)	9 (39.1)
**RECIST disease progression, n (%)**	32 (36.8)	8 (38.1)	11 (52.4)	4 (18.2)	9 (39.1)
**Died, n (%)**	10 (11.5)	2 (9.5)	4 (19.0)	4 (18.2)	0
**Not evaluable, n (%)**	1 (1.1)	0	0	1 (4.5)	0
**Disease control at 12 weeks, n (%)**	32 (36.8)	8 (38.1)	2 (9.5)	10 (45.5)	12 (52.2)
**Disease control at 24 weeks, n (%)**	15 (17.2)	4 (19.0)	2 (9.5)	3 (13.6)	6 (26.1)
**PFS, median (80% CI), months**	2.7 (1.6-3.0)	2.8 (1.4-5.3)	1.4 (1.4-1.8)	3.4 (2.1-4.9)	4.2 (2.7-4.4)
**OS, median (80% CI), months**	9.4 (6.9-10.8)	9.6 (5.3-16.0)	5.8 (5.3-10.8)	7.2 (4.9-10.3)	15.5 (9.4-20.9)

CI, confidence interval; OS, overall survival; PFS, progression-free survival; RECIST, Response Evaluation Criteria In Solid Tumours.

Supplementary Table 4 | Treatment efficacy with durvalumab-danvatirsen, by cohort.

		**Biomarker-non-matched**	
**Efficacy parameter**	**All, *n* = 45**	**Primary, *n* = 23**	**Acquired, *n* = 22**
**Objective response rate, n (%)**	0	0	0
**Partial response rate, n (%)**	0	0	0
**Stable disease ≥40 days, n (%)**	25 (55.6)	11 (47.8)	14 (63.6)
**Unconfirmed partial or complete response, n (%)**	0	0	0
**Progression, n (%)**	18 (40.0)	11 (47.8)	7 (31.8)
**RECIST disease progression, n (%)**	14 (31.1)	9 (39.1)	5 (22.7)
**Died, n (%)**	4 (8.9)	2 (8.7)	2 (9.1)
**Not evaluable, n (%)**	2 (4.4)	1 (4.3)	1 (4.5)
**Disease control at 12 weeks, n (%)**	12 (26.7)	3 (13.0)	9 (40.9)
**Disease control at 24 weeks, n (%)**	6 (13.3)	0	6 (27.3)
**PFS, median (80% CI), months**	2.9 (1.7–3.1)	1.7 (1.6-3.0)	3.1 (2.8-6.1)
**OS, median (80% CI), months**	7.9 (6.0–10.6)	6.0 (3.6-6.5)	11.2 (9.7-12.6)

CI, confidence interval; OS, overall survival; PFS, progression-free survival; RECIST, Response Evaluation Criteria In Solid Tumours.

Supplementary Table 5 | Treatment efficacy with durvalumab-oleclumab, by cohort.

		Biomarker-matched	Biomarker-non-matched	
**Efficacy parameter**	**All, *n* = 57**	**CD73 high cohort, *n* = 23**	**Primary, *n* = 9**	**Acquired, *n* = 25**
**Objective response rate, n (%)**	1 (1.8)	0	0	1 (4.0)
**Partial response rate, n (%)**	1 (1.8)	0	0	1 (4.0)
**Stable disease ≥35 days, n (%)**	24 (42.1)	8 (34.8)	2 (22.2)	14 (56.0)
**Unconfirmed partial or complete response, n (%)**	3 (5.3)	1 (4.3)	0	2 (8.0)
**Progression, n (%)**	31 (54.4)	14 (60.9)	7 (77.8)	10 (40.0)
**RECIST disease progression, n (%)**	24 (42.1)	11 (47.8)	5 (55.6)	8 (32.0)
**Died, n (%)**	7 (12.3)	3 (13.0)	2 (22.2)	2 (8.0)
**Not evaluable, n (%)**	1 (1.8)	1 (4.3)	0	0
**Disease control at 12 weeks, n (%)**	17 (29.8)	7 (30.4)	1 (11.1)	9 (36.0)
**Disease control at 24 weeks, n (%)**	9 (15.8)	2 (8.7)	1 (11.1)	6 (24.0)
**PFS, median (80% CI), months**	1.8 (1.6-2.7)	1.6 (1.4-2.8)	1.4 (1.4-1.8)	2.7 (1.7-4.2)
**OS, median (80% CI), months**	11.0 (7.6-13.5)	11.0 (7.6-15.7)	7.1 (4.9-12.0)	12.8 (7.4-21.0)

CI, confidence interval; OS, overall survival; PFS, progression-free survival; RECIST, Response Evaluation Criteria In Solid Tumours.

Supplementary Table 6 | Summary of safety profile of durvalumab-ceralasertib, by cohort

		Biomarker-matched	Biomarker-non-matched	
**AE category, n (%)**	**All, *n* = 79**	**ATM cohort, *n* = 23**	**Primary, *n* = 23**	**Acquired, *n* = 33**
**Any TEAE**	74 (93.7)	23 (100)	20 (87.0)	31 (93.9)
**Any TRAE**	60 (75.9)	22 (95.7)	16 (69.6)	22 (66.7)
**Related to durvalumab only**	21 (26.6)	9 (39.1)	6 (26.1)	6 (18.2)
**Related to ceralasertib only**	53 (67.1)	18 (78.3)	13 (56.5)	22 (66.7)
**Related to both**	23 (29.1)	10 (43.5)	6 (26.1)	7 (21.2)
**Any grade ≥3 TEAE**	35 (44.3)	11 (47.8)	8 (34.8)	16 (48.5)
**Any grade ≥3 TRAE**	16 (20.3)	7 (30.4)	2 (8.7)	7 (21.2)
**Related to durvalumab only**	2 (2.5)	2 (8.7)	0	0
**Related to ceralasertib only**	14 (17.7)	6 (26.1)	1 (4.3)	7 (21.2)
**Related to both**	6 (7.6)	3 (13.0)	1 (4.3)	2 (6.1)
**Any TEAE with an outcome of death**	2 (2.5)	0	0	2 (6.1)
**Any SAE**	29 (36.7)	10 (43.5)	7 (30.4)	12 (36.4)
**Any SAE related to any treatment**	10 (12.7)	6 (26.1)	2 (8.7)	2 (6.1)
**Related to durvalumab only**	2 (2.5)	2 (8.7)	0	0
**Related to ceralasertib only**	5 (6.3)	3 (13.0)	1 (4.3)	1 (3.0)
**Related to both**	3 (3.8)	1 (4.3)	1 (4.3)	1 (3.0)
**Any TEAE leading to discontinuation of any treatment**	9 (11.4)	4 (17.4)	3 (13.0)	2 (6.1)

AE, adverse event; SAE, serious adverse event; TEAE, treatment-emergent adverse event; TRAE, treatment-related adverse event.

Supplementary Table 7 | Summary of safety profile of durvalumab-olaparib, by cohort

		Biomarker-matched		Biomarker-non-matched	
**AE category, n (%)**	**All, *n* = 87**	**HRRm cohort, *n* = 21**	***STK11/LKB1* cohort, *n* = 21**	**Primary, *n* = 22**	**Acquired, *n* = 23**
**Any TEAE**	80 (92.0)	20 (95.2)	19 (90.5)	20 (90.9)	21 (91.3)
**Any TRAE**	67 (77.0)	16 (76.2)	15 (71.4)	16 (72.7)	20 (87.0)
**Related to durvalumab only**	24 (27.6)	8 (38.1)	4 (19.0)	4 (18.2)	8 (34.8)
**Related to olaparib only**	53 (60.9)	14 (66.7)	9 (42.9)	13 (59.1)	17 (73.9)
**Related to both**	29 (33.3)	9 (42.9)	5 (23.8)	7 (31.8)	8 (34.8)
**Any grade ≥3 TEAE**	47 (54.0)	15 (74.1)	8 (38.1)	12 (54.5)	12 (52.2)
**Any grade ≥3 TRAE**	30 (34.5)	10 (47.6)	3 (14.3)	9 (40.9)	8 (34.8)
**Related to durvalumab only**	8 (9.2)	4 (19.0)	1 (4.8)	1 (4.5)	2 (8.7)
**Related to olaparib only**	18 (20.7)	5 (23.8)	2 (9.5)	6 (27.3)	5 (21.7)
**Related to both**	8 (9.2)	3 (14.3)	0	2 (9.1)	3 (13.0)
**Any TEAE with an outcome of death**	1 (1.1)	1 (4.8)	0	0	0
**Any SAE**	31 (35.6)	9 (42.9)	7 (33.3)	8 (36.4)	7 (30.4)
**Any SAE related to any treatment**	9 (10.3)	4 (19.0)	0	5 (22.7)	0
**Related to durvalumab only**	5 (5.7)	4 (19.0)	0	1 (4.5)	0
**Related to olaparib only**	2 (2.3)	0	0	2 (9.1)	0
**Related to both**	4 (4.6)	2 (9.5)	0	2 (9.1)	0
**Any TEAE leading to discontinuation of any treatment**	8 (9.2)	5 (23.8)	1 (4.8)	0	2 (8.7)

AE, adverse event; SAE, serious adverse event; TEAE, treatment-emergent adverse event; TRAE, treatment-related adverse event.

Supplementary Table 8 | Summary of safety profile of durvalumab-danvatirsen, by cohort

		Biomarker-non-matched	
**AE category, n (%)**	**All, *n* = 45**	**Primary, *n* = 23**	**Acquired, *n* = 22**
**Any TEAE**	43 (95.6)	23 (100)	20 (90.9)
**Any TRAE**	33 (73.3)	16 (69.6)	17 (77.3)
**Related to durvalumab only**	11 (24.4)	6 (26.1)	5 (22.7)
**Related to combination agent only**	16 (35.6)	8 (34.8)	8 (36.4)
**Related to both**	21 (46.7)	8 (34.8)	13 (59.1)
**Any grade ≥3 TEAE**	28 (62.2)	13 (56.5)	15 (68.2)
**Any grade ≥3 TRAE**	17 (37.8)	5 (21.7)	12 (54.5)
**Related to durvalumab only**	3 (6.7)	1 (4.3)	2 (9.1)
**Related to combination agent only**	5 (11.1)	1 (4.3)	4 (18.2)
**Related to both**	9 (20.0)	3 (13.0)	6 (27.3)
**Any TEAE with an outcome of death**	3 (6.7)	2 (8.7)	1 (4.5)
**Any SAE**	20 (44.4)	12 (52.2)	8 (36.4)
**Any SAE related to any treatment**	3 (6.7)	1 (4.3)	2 (9.1)
**Related to durvalumab only**	0	0	0
**Related to combination agent only**	1 (2.2)	0	1 (4.5)
**Related to both**	2 (4.4)	1 (4.3)	1 (4.5)
**Any TEAE leading to discontinuation of any treatment**	10 (22.2)	5 (21.7)	5 (22.7)

AE, adverse event; SAE, serious adverse event; TEAE, treatment-emergent adverse event; TRAE, treatment-related adverse event.

Supplementary Table 9 | Summary of safety profile of durvalumab-oleclumab, by cohort

		Biomarker-matched	Biomarker-non-matched	
**AE category, n (%)**	**All, *n* = 57**	**CD73 high cohort, *n* = 23**	**Primary, *n* = 9**	**Acquired, *n* = 25**
**Any TEAE**	47 (82.5)	21 (91.3)	5 (55.6)	21 (84.0)
**Any TRAE**	35 (61.4)	15 (65.2)	4 (44.4)	16 (64.0)
**Related to durvalumab only**	14 (24.6)	3 (13.0)	2 (22.2)	9 (36.0)
**Related to oleclumab only**	6 (10.5)	3 (13.0)	1 (11.1)	2 (8.0)
**Related to both**	24 (42.1)	11 (47.8)	3 (33.3)	10 (40.0)
**Any grade ≥3 TEAE**	22 (38.6)	10 (43.5)	3 (33.3)	9 (36.0)
**Any grade ≥3 TRAE**	10 (17.5)	5 (21.7)	2 (22.2)	3 (12.0)
**Related to durvalumab only**	2 (3.5)	1 (4.3)	0	1 (4.0)
**Related to oleclumab only**	3 (5.3)	1 (4.3)	1 (11.1)	1 (4.0)
**Related to both**	6 (10.5)	3 (13.0)	1 (11.1)	2 (8.0)
**Any TEAE with an outcome of death**	1 (1.8)	0	0	1 (4.0)
**Any SAE**	14 (24.6)	8 (34.8)	1 (11.1)	5 (20.0)
**Any SAE related to any treatment**	5 (8.8)	3 (13.0)	1 (11.1)	1 (4.0)
**Related to durvalumab only**	1 (1.8)	1 (4.3)	0	0
**Related to oleclumab only**	3 (5.3)	1 (4.3)	1 (11.1)	1 (4.0)
**Related to both**	2 (3.5)	1 (4.3)	0	1 (4.0)
**Any TEAE leading to discontinuation of any treatment**	7 (12.3)	3 (13.0)	0	4 (16.0)

AE, adverse event; SAE, serious adverse event; TEAE, treatment-emergent adverse event; TRAE, treatment-related adverse event.

Supplementary Table 10 | Patient baseline characteristics in biomarker-evaluable population for gene expression analysis by RNA sequencing.

Characteristic	Durvalumab plus olaparib, danvatirsen or oleclumab, *n* = 49
**Age, median (range), years**	64.0 (48–85)
**Age <65 years, n (%)**	25 (51.0)
**Age ≥65 years, n (%)**	24 (49.0)
**Male sex, n (%)[a]**	19 (38.8)
**Race, n (%)**	
**White**	44 (89.8)
**Asian**	2 (4.1)
**Black or African American**	3 (6.1)
**Native Hawaiian or Other Pacific Islander**	0
**Other[b]**	0
**ECOG PS, n (%)**	
**0**	17 (34.7)
**1**	32 (65.3)
**Histology, n (%)**	
**Adenocarcinoma**	38 (77.6)
**Squamous cell carcinoma**	6 (12.2)
**Large-cell carcinoma (NOS)**	1 (2.0)
**Other**	4 (18.2)
**Time from diagnosis, n (%)[c]**	
**≤12 months**	12 (24.5)
**>12 months**	36 (73.5)
**Missing**	1 (2.0)
**Disease classification, n (%)[c]**	
**Metastatic**	48 (98.0)
**Locally advanced**	0
**Missing**	1 (2.0)
**Metastatic sites, n (%)**	
**≤2**	35 (71.4)
**≥3**	14 (28.6)
**Metastatic site location, n (%)**	
**Bone and locomotor**	14 (28.6)
**Adrenal gland**	13 (26.5)
**Brain/CNS and/or other CNS**	9 (18.4)
**Liver and/or hepatic (including gall bladder)**	10 (20.4)
**PD-L1 status, n (%)**	
**Positive (TC ≥1%)**	24 (49.0)
**1–49%**	15 (30.6)
**≥50%**	9 (18.4)
**Negative (TC <1%)**	8 (16.3)

Characteristic	Durvalumab plus olaparib, danvatirsen or oleclumab, *n* = 49
Unknown	17 (34.7)
Prior regimens, n (%)	
1	7 (14.3)
2	27 (55.1)
3	10 (20.4)
≥4	5 (10.2)
Prior immunotherapies, n (%)	
1	49 (100)
Prior anti-PD-(L)1 immunotherapy, n (%)	
Nivolumab	19 (38.8)
Pembrolizumab	19 (38.8)
Durvalumab	6 (12.2)
Atezolizumab	5 (10.2)
Prior anti-CTLA4 immunotherapy, n (%)	
Tremelimumab	2 (4.1)
Ipilimumab	1 (2.0)
Best response on prior immunotherapy, n (%)	
Complete response	1 (2.0)
Partial response	10 (20.4)
Stable disease	19 (38.8)
Stable disease, biomarker-matched patients	8 (16.3)
Stable disease, biomarker-non-matched patients	11 (22.4)
Progressive disease	16 (32.7)
Non-evaluable	3 (6.1)
Median time from prior immunotherapy, months (range)	4.6 (0.9–50.1)
Prior immunotherapy and resistance classification	
Primary resistance, n (%)	18 (36.7)
Prior monotherapy	14 (28.6)
Prior combination	4 (8.2)
Both monotherapy and combination	0
Acquired resistance, n (%)	31 (63.3)
Prior monotherapy	21 (42.9)
Prior combination	9 (18.4)
Both monotherapy and combination	1 (2.0)
Prior platinum-based therapies, n (%)	
1	43 (87.8)
2	5 (10.2)
≥3	1 (2.0)
Smoking status, n (%)	
Never	8 (16.3)
Current	7 (14.3)

Characteristic	Durvalumab plus olaparib, danvatirsen or oleclumab, *n* = 49
Former	34 (69.4)
**Neutrophil count, n (%)**	
<10 x 10^9 cells/L	47 (95.9)
≥10 x 10^9 cells/L	2 (4.1)
**Platelet count, n (%)**	
<450 x 10^9 cells/L	42 (85.7)
≥450 x 10^9 cells/L	7 (14.3)
**Haemoglobin level, n (%)**	
>10 g/dL	49 (100)
≤10 g/dL	0
**C-reactive protein, n (%)**	
> overall median[d]	23 (46.9)
≤ overall median[d]	25 (51.0)
**Lymphocyte count, n (%)**	
> overall median[d]	22 (44.9)
≤ overall median[d]	27 (55.1)
**Lactate dehydrogenase level, n (%)**	
> overall median[d]	23 (46.9)
≤ overall median[d]	26 (53.1)

[a]Sex as recorded by investigator in the case report form. [b]Recorded as 'Other' in the case report form. [c]For parameters for which data are missing, the numbers of patients with data are indicated for each regimen and are used as the denominators for calculating percentages. [d]Overall median values for all patients within the respective study modules.

CNS, central nervous system; ECOG PS, Eastern Cooperative Oncology Group performance status; NOS, not otherwise specified; PD-L1, programmed cell death ligand 1.

Supplementary Table 11 | Patient baseline characteristics in patients receiving durvalumab-ceralasertib or durvalumab plus olaparib, danvatirsen or oleclumab, by prior anti-PD-L1 therapy resistance status (primary, acquired), pooled across biomarker-matched and biomarker-non-matched cohorts.

Characteristic	Primary resistance, *n* = 111		Acquired resistance, *n* = 157	
	Durvalumab-ceralasertib, *n* = 30	Durvalumab plus olaparib, danvatirsen or oleclumab, *n* = 81	Durvalumab-ceralasertib, *n* = 49	Durvalumab plus olaparib, danvatirsen or oleclumab, *n* = 108
**Age, median (range), years**	61.0 (42-76)	62.0 (35-80)	64.0 (42-80)	64.0 (37-85)
**Age <65 years, n (%)**	20 (66.7)	45 (55.6)	25 (51.0)	57 (52.8)
**Age ≥65 years, n (%)**	10 (33.3)	36 (44.4)	24 (49.0)	51 (47.2)
**Male sex, n (%)[a]**	22 (73.3)	43 (53.1)	30 (61.2)	60 (55.6)
**Race, n (%)[b]**	*n* = 30	*n* = 80	*n* = 47	*n* = 107
**White**	17 (56.7)	58 (72.5)	30 (63.8)	66 (61.7)
**Asian**	6 (20.0)	9 (11.3)	3 (6.4)	29 (27.1)
**Black or African American**	1 (3.3)	2 (2.5)	2 (4.3)	2 (1.9)
**Native Hawaiian or Other Pacific Islander**	0	1 (1.3)	0	0
**Other[c]**	6 (20.0)	10 (12.5)	12 (25.5)	10 (9.3)
**ECOG PS, n (%)[b]**	*n* = 30	*n* = 80	*n* = 49	*n* = 108
**0**	7 (23.3)	28 (35.0)	21 (42.9)	36 (33.3)
**1**	23 (76.7)	52 (65.0)	28 (57.1)	71 (65.7)
**2[d]**	0	0	0	1 (0.9)
**Histology, n (%)**				
**Adenocarcinoma**	18 (60.0)	58 (71.6)	37 (75.5)	73 (67.6)
**Squamous cell carcinoma**	10 (33.3)	19 (23.5)	9 (18.4)	24 (22.2)
**Large-cell carcinoma (NOS)**	1 (3.3)	1 (1.2)	1 (2.0)	5 (4.6)
**Other**	1 (3.3)	3 (3.7)	2 (4.1)	6 (5.6)
**Time from diagnosis, n (%)[b]**	*n* = 30	*n* = 79	*n* = 49	*n* = 105
**≤12 months**	11 (36.7)	32 (40.5)	2 (4.1)	12 (11.4)
**>12 months**	19 (63.3)	47 (59.5)	47 (95.9)	93 (88.6)

Characteristic	Primary resistance, *n* = 111		Acquired resistance, *n* = 157	
	**Durvalumab-ceralasertib, *n* = 30**	**Durvalumab plus olaparib, danvatirsen or oleclumab, *n* = 81**	**Durvalumab-ceralasertib, *n* = 49**	**Durvalumab plus olaparib, danvatirsen or oleclumab, *n* = 108**
**Disease classification, n (%)[b]**	*n* = 30	*n* = 81	*n* = 49	*n* = 107
**Metastatic**	28 (93.3)	80 (98.8)	49 (100)	104 (97.2)
**Locally advanced**	2 (6.7)	1 (1.2)	0	3 (2.8)
**Metastatic sites, n (%)**				
**≤2**	10 (33.3)	50 (61.7)	21 (42.9)	62 (57.4)
**≥3**	20 (66.7)	31 (38.3)	28 (57.1)	46 (42.6)
**Metastatic site location, n (%)**				
**Bone and locomotor**	9 (30.0)	18 (22.2)	15 (30.6)	37 (34.3)
**Adrenal gland**	6 (20.0)	15 (18.5)	9 (18.4)	22 (20.4)
**Brain/CNS and/or other CNS**	4 (13.3)	17 (21.0)	8 (16.3)	19 (17.6)
**Liver and/or hepatic (including gall bladder)**	6 (20.0)	18 (22.2)	8 (16.3)	17 (15.7)
**PD-L1 status, n (%)**				
**Positive (TC ≥1%)**	16 (53.3)	37 (45.7)	26 (53.1)	48 (44.4)
**1–49%**	11 (36.7)	25 (30.9)	14 (28.6)	21 (19.4)
**≥50%**	5 (16.7)	12 (14.8)	12 (24.5)	27 (25.0)
**Negative (TC <1%)**	11 (36.7)	17 (21.0)	10 (20.4)	13 (12.0)
**Unknown**	3 (10.0)	27 (33.3)	13 (26.5)	46 (42.6)
**Missing**	0	0	0	1 (0.9)
**Prior regimens, n (%)**				
**1**	5 (16.7)	8 (9.9)	10 (20.4)	14 (13.0)
**2**	8 (26.7)	37 (45.7)	19 (38.8)	48 (44.4)
**3**	13 (43.3)	22 (27.2)	9 (18.4)	25 (23.1)
**≥4**	4 (13.3)	14 (17.3)	11 (22.4)	21 (19.4)
**Prior immunotherapies, n (%)**				
**1**	29 (96.7)	80 (98.8)	48 (98.0)	108 (100)

Characteristic	Primary resistance, *n* = 111		Acquired resistance, *n* = 157	
	**Durvalumab-ceralasertib, *n* = 30**	**Durvalumab plus olaparib, danvatirsen or oleclumab, *n* = 81**	**Durvalumab-ceralasertib, *n* = 49**	**Durvalumab plus olaparib, danvatirsen or oleclumab, *n* = 108**
**2**	1 (3.3)	1 (1.2)	1 (2.0)	0
**Prior anti-PD-(L)1 immunotherapy, n (%)**				
**Nivolumab**	12 (40.0)	38 (46.9)	24 (49.0)	55 (50.9)
**Pembrolizumab**	15 (50.0)	22 (27.2)	18 (36.7)	30 (27.8)
**Atezolizumab**	2 (6.7)	13 (16.0)	5 (10.2)	13 (12.0)
**Durvalumab**	0	8 (9.9)	1 (2.0)	9 (8.3)
**Cemiplimab**	0	0	0	1 (0.9)
**Prior anti-CTLA4 immunotherapy, n (%)**				
**Tremelimumab**	0	2 (2.5)	1 (2.0)	3 (2.8)
**Ipilimumab**	0	2 (2.5)	0	0
**Best response on prior immunotherapy, n (%)**				
**Complete response**	0	0	0	2 (1.9)
**Partial response**	0	10 (12.3)	17 (34.7)	44 (40.7)
**Stable disease**	15 (50.0)	20 (24.7)	25 (51.0)	44 (40.7)
**Stable disease, biomarker-matched patients**	4 (13.3)	9 (11.1)	7 (14.3)	18 (16.7)
**Stable disease, biomarker-non-matched patients**	11 (36.7)	11 (13.6)	18 (36.7)	26 (24.1)
**Progressive disease**	12 (40.0)	45 (55.6)	4 (8.2)	12 (11.1)
**Non-evaluable**	1 (3.3)	4 (4.9)	2 (4.1)	6 (5.6)
**Not applicable**	1 (3.3)	1 (1.2)	0	0
**Time from prior immunotherapy**	*n* = 29	*n* = 80	*n* = 48	*n* = 108
**Median, months (range)**	3.9 (1.6–30.6)	3.2 (0.7–33.3)	3.9 (0.7–31.4)	3.2 (0.8–50.1)
**Prior immunotherapy and resistance classification**				
**Primary resistance, n (%)**	29 (96.7)	80 (98.8)	0	0
**Prior monotherapy**	22 (73.3)	63 (77.8)	0	0
**Prior combination**	5 (16.7)	16 (19.8)	0	0

Characteristic	Primary resistance, *n* = 111		Acquired resistance, *n* = 157	
	Durvalumab-ceralasertib, *n* = 30	Durvalumab plus olaparib, danvatirsen or oleclumab, *n* = 81	Durvalumab-ceralasertib, *n* = 49	Durvalumab plus olaparib, danvatirsen or oleclumab, *n* = 108
Both monotherapy and combination	2 (6.7)	1 (1.2)	0	0
Acquired resistance, n (%)	0	0	48 (98.0)	108 (100)
Prior monotherapy	0	0	36 (73.5)	85 (78.7)
Prior combination	0	0	7 (14.3)	18 (16.7)
Both monotherapy and combination	0	0	5 (10.2)	5 (4.6)
Not available, n (%)	1 (3.3	1 (1.2)	1 (2.0)	0
Prior platinum-based therapies, n (%)				
1	22 (73.3)	65 (80.2)	40 (81.6)	89 (82.4)
2	8 (26.7)	12 (14.8)	7 (14.3)	16 (14.8)
≥3	0	4 (4.9)	2 (4.1)	3 (2.8)
Smoking status, n (%)				
Never	2 (6.7)	14 (17.3)	7 (14.3)	14 (13.0)
Current	4 (13.3)	7 (8.6)	14 (28.6)	16 (14.8)
Former	24 (80.0)	60 (74.1)	28 (57.1)	78 (72.2)

[a]Sex as recorded by investigator in the case report form. [b]For parameters for which data are missing, the numbers of patients with data are indicated for each regimen and are used as the denominators for calculating percentages. [c]Recorded as 'Other' in the case report form. [d]Patient did not meet the eligibility criterion for ECOG PS of 0–1.

CNS, central nervous system; ECOG PS, Eastern Cooperative Oncology Group performance status; NOS, not otherwise specified; PD-L1, programmed cell death ligand 1.

Supplementary Table 12 | Patient baseline characteristics in patients receiving durvalumab-ceralasertib or durvalumab plus olaparib, danvatirsen or oleclumab, by histology (squamous, non-squamous).

Characteristic	Squamous, *n* = 62		Non-squamous, *n* = 206	
	Durvalumab-ceralasertib, *n* = 19	Durvalumab plus olaparib, danvatirsen or oleclumab, *n* = 43	Durvalumab-ceralasertib, *n* = 60	Durvalumab plus olaparib, danvatirsen or oleclumab, *n* = 146
**Age, median (range), years**	64.0 (42-74)	65.0 (39-82)	63.0 (42-80)	63.0 (35-85)
**Age <65 years, n (%)**	10 (52.6)	21 (48.8)	35 (58.3)	81 (55.5)
**Age ≥65 years, n (%)**	9 (47.4)	22 (51.2)	25 (41.7)	65 (44.5)
**Male sex, n (%)[a]**	16 (84.2)	32 (74.4)	36 (60.0)	71 (48.6)
**Race, n (%)[b]**	*n* = 18	*n* = 43	*n* = 59	*n* = 144
**White**	9 (50.0)	22 (51.2)	38 (64.4)	102 (70.8)
**Asian**	4 (22.2)	16 (37.2)	5 (8.5)	22 (15.3)
**Black or African American**	1 (5.6)	0	2 (3.4)	4 (2.8)
**Native Hawaiian or Other Pacific Islander**	0	0	0	1 (0.7)
**Other[c]**	4 (22.2)	5 (11.6)	14 (23.7)	15 (10.4)
**ECOG PS, n (%)[b]**	*n* = 19	*n* = 42	*n* = 60	*n* = 146
**0**	6 (31.6)	12 (28.6)	22 (36.7)	52 (35.6)
**1**	13 (68.4)	30 (71.4)	38 (63.3)	93 (63.7)
**2[d]**	0	0	0	1 (0.7)
**Histology, n (%)**				
**Adenocarcinoma**	0	0	55 (91.7)	131 (89.7)
**Squamous cell carcinoma**	19 (100)	43 (100)	0	0
**Large-cell carcinoma (NOS)**	0	0	2 (3.3)	6 (4.1)
**Other**	0	0	3 (5.0)	9 (6.2)
**Time from diagnosis, n (%)[b]**	*n* = 19	*n* = 41	*n* = 60	*n* = 143
**≤12 months**	4 (21.1)	10 (24.4)	9 (15.0)	34 (23.8)
**>12 months**	15 (78.9)	31 (75.6)	51 (85.0)	109 (76.2)
**Disease classification, n (%)[b]**	*n* = 19	*n* = 43	*n* = 60	*n* = 145

Characteristic	Squamous, *n* = 62		Non-squamous, *n* = 206	
	Durvalumab-ceralasertib, *n* = 19	Durvalumab plus olaparib, danvatirsen or oleclumab, *n* = 43	Durvalumab-ceralasertib, *n* = 60	Durvalumab plus olaparib, danvatirsen or oleclumab, *n* = 146
Metastatic	17 (89.5)	41 (95.3)	60 (100)	143 (97.9)
Locally advanced	2 (10.5)	2 (4.7)	0	2 (1.4)
Metastatic sites, n (%)				
≤2	8 (42.1)	25 (58.1)	23 (38.3)	87 (59.6)
≥3	11 (57.9)	18 (41.9)	37 (61.7)	59 (40.4)
Metastatic site location, n (%)				
Bone and locomotor	2 (10.5)	12 (27.9)	22 (36.7)	43 (29.5)
Adrenal gland	2 (10.5)	5 (11.6)	13 (21.7)	32 (21.9)
Brain/CNS and/or other CNS	2 (10.5)	4 (9.3)	10 (16.7)	32 (21.9)
Liver and/or hepatic (including gall bladder)	3 (15.8)	6 (14.0)	11 (18.3)	29 (19.9)
PD-L1 status, n (%)				
Positive (TC ≥1%)	11 (57.9)	20 (46.5)	31 (51.7)	65 (44.5)
1–49%	6 (31.6)	12 (27.9)	19 (31.7)	34 (23.3)
≥50%	5 (26.3)	8 (18.6)	12 (20.0)	31 (21.2)
Negative (TC <1%)	6 (31.6)	6 (14.0)	15 (25.0)	24 (16.4)
Unknown	2 (10.5)	17 (39.5)	14 (23.3)	56 (38.4)
Missing	0	0	0	1 (0.7)
Prior regimens, n (%)				
1	2 (10.5)	2 (4.7)	13 (21.7)	20 (13.7)
2	7 (36.8)	19 (44.2)	20 (33.3)	66 (45.2)
3	7 (36.8)	15 (34.9)	15 (25.0)	32 (21.9)
≥4	3 (15.8)	7 (16.3)	12 (20.0)	28 (19.2)
Prior immunotherapies, n (%)				
1	19 (100)	42 (97.7)	58 (96.7)	146 (100)
2	0	1 (2.3)	2 (3.3)	0

Characteristic	Squamous, *n* = 62		Non-squamous, *n* = 206	
	**Durvalumab-ceralasertib, *n* = 19**	**Durvalumab plus olaparib, danvatirsen or oleclumab, *n* = 43**	**Durvalumab-ceralasertib, *n* = 60**	**Durvalumab plus olaparib, danvatirsen or oleclumab, *n* = 146**
**Prior anti-PD-(L)1 immunotherapy, n (%)**				
**Nivolumab**	9 (47.4)	25 (58.1)	27 (45.0)	68 (46.6)
**Pembrolizumab**	8 (42.1)	7 (16.3)	25 (41.7)	45 (30.8)
**Atezolizumab**	1 (5.3)	8 (18.6)	6 (10.0)	18 (12.3)
**Durvalumab**	0	3 (7.0)	1 (1.7)	14 (9.6)
**Cemiplimab**	0	1 (2.3)	0	0
**Prior anti-CTLA4 immunotherapy, n (%)**				
**Tremelimumab**	0	0	1 (1.7)	5 (3.4)
**Ipilimumab**	0	1 (2.3)	0	1 (0.7)
**Best response on prior immunotherapy, n (%)**				
**Complete response**	0	0	0	2 (1.4)
**Partial response**	2 (10.5)	16 (37.2)	15 (25.0)	38 (26.0)
**Stable disease**	10 (52.6)	9 (20.9)	30 (50.0)	55 (37.7)
**Stable disease, biomarker-matched patients**	0	3 (7.0)	11 (18.3)	24 (16.4)
**Stable disease, biomarker-non-matched patients**	10 (52.6)	6 (14.0)	19 (31.7)	31 (21.2)
**Progressive disease**	5 (26.3)	13 (30.2)	11 (18.3)	44 (30.1)
**Non-evaluable**	1 (5.3)	4 (9.3)	2 (3.3)	6 (4.1)
**Not applicable**	0	1 (2.3)	1 (1.7)	0
**Time from prior immunotherapy**	*n* = 18	*n* = 43	*n* = 59	*n* = 145
**Median, months (range)**	5.1 (0.8–31.4)	3.2 (0.7–37.9)	3.5 (0.7–25.3)	3.2 (0.8–50.1)
**Prior immunotherapy and resistance classification**				
**Primary resistance, n (%)**	10 (52.6)	19 (44.2)	19 (31.7)	61 (41.8)
**Prior monotherapy**	9 (47.4)	18 (41.9)	13 (21.7)	45 (30.8)
**Prior combination**	1 (5.3)	1 (2.3)	4 (6.7)	15 (10.3)

Characteristic	Squamous, *n* = 62		Non-squamous, *n* = 206	
	**Durvalumab-ceralasertib, *n* = 19**	**Durvalumab plus olaparib, danvatirsen or oleclumab, *n* = 43**	**Durvalumab-ceralasertib, *n* = 60**	**Durvalumab plus olaparib, danvatirsen or oleclumab, *n* = 146**
Both monotherapy and combination	0	0	2 (3.3)	1 (0.7)
Acquired resistance, n (%)	8 (42.1)	24 (55.8)	40 (66.7)	84 (57.5)
Prior monotherapy	7 (36.8)	22 (51.2)	29 (48.3)	63 (43.2)
Prior combination	1 (5.3)	1 (2.3)	6 (10.0)	17 (11.6)
Both monotherapy and combination	0	1 (2.3)	5 (8.3)	4 (2.7)
Not available, n (%)	1 (5.3)	0	1 (1.7)	1 (0.7)
Prior platinum-based therapies, n (%)				
1	14 (73.7)	34 (79.1)	48 (80.0)	120 (82.2)
2	5 (26.3)	8 (18.6)	10 (16.7)	20 (13.7)
≥3	0	1 (2.3)	2 (3.3)	6 (4.1)
Smoking status, n (%)				
Never	5 (26.3)	7 (16.3)	4 (6.7)	21 (14.4)
Current	2 (10.5)	3 (7.0)	16 (26.7)	20 (13.7)
Former	12 (63.2)	33 (76.7)	40 (66.7)	105 (71.9)

[a]Sex as recorded by investigator in the case report form. [b]For parameters for which data are missing, the numbers of patients with data are indicated for each regimen and are used as the denominators for calculating percentages. [c]Recorded as 'Other' in the case report form. [d]Patient did not meet the eligibility criterion for ECOG PS of 0–1.

CNS, central nervous system; ECOG PS, Eastern Cooperative Oncology Group performance status; NOS, not otherwise specified; PD-L1, programmed cell death ligand 1.

Supplementary Table 13 | Patient baseline characteristics in biomarker-evaluable population for peripheral gene expression analysis (GEA) in those receiving durvalumab-ceralasertib and for T-cell receptor repertoire (TCR) analysis in those receiving durvalumab-ceralasertib or durvalumab plus olaparib or danvatirsen.

Characteristic	Peripheral GEA	TCR analysis	
	**Durvalumab-ceralasertib, *n* = 48**	**Durvalumab-ceralasertib, *n* = 64**	**Durvalumab plus olaparib or danvatirsen, *n* = 123**
**Age, median (range), years**	63.0 (45–80)	64.0 (45–80)	64.0 (35–85)
**Age <65 years, n (%)**	28 (58.3)	35 (54.7)	65 (52.8)
**Age ≥65 years, n (%)**	20 (41.7)	29 (45.3)	58 (47.2)
**Male sex, n (%)[a]**	32 (66.7)	43 (67.2)	66 (53.7)
**Race, n (%)[b]**	*n* = 46	*n* = 62	*n* = 121
**White**	31 (67.4)	41 (66.1)	79 (65.3)
**Asian**	5 (10.9)	8 (12.9)	29 (24.0)
**Black or African American**	0	1 (1.6)	4 (3.3)
**Other[c]**	10 (21.7)	12 (19.4)	9 (7.4)
**ECOG PS, n (%)**			
**0**	18 (37.5)	23 (35.9)	39 (31.7)
**1**	30 (62.5)	41 (64.1)	83 (67.5)
**2[d]**	0	0	1 (0.8)
**Histology, n (%)**			
**Adenocarcinoma**	35 (72.9)	43 (67.2)	90 (73.2)
**Squamous cell carcinoma**	11 (22.9)	16 (25.0)	26 (21.1)
**Large-cell carcinoma (NOS)**	1 (2.1)	2 (3.1)	4 (3.3)
**Other**	1 (2.1)	3 (4.7)	3 (2.4)
**Time from diagnosis, n (%)[b]**	*n* = 48	*n* = 64	*n* = 119
**≤12 months**	9 (18.8)	11 (17.2)	30 (25.2)
**>12 months**	39 (81.3)	53 (82.8)	89 (74.8)
**Disease classification, n (%)[b]**	*n* = 48	*n* = 64	*n* = 122
**Metastatic**	47 (97.9)	62 (96.9)	121 (99.2)

Characteristic	Peripheral GEA	TCR analysis	
	Durvalumab-ceralasertib, *n* = 48	Durvalumab-ceralasertib, *n* = 64	Durvalumab plus olaparib or danvatirsen, *n* = 123
Locally advanced	1 (2.1)	2 (3.1)	1 (0.8)
**Metastatic sites, n (%)**			
≤2	18 (37.5)	27 (42.2)	72 (58.5)
≥3	30 (62.5)	37 (57.8)	51 (41.5)
**Metastatic site location, n (%)**			
Bone and locomotor	14 (29.2)	19 (29.7)	36 (29.3)
Adrenal gland	7 (14.6)	11 (17.2)	25 (20.3)
Brain/CNS and/or other CNS	9 (18.8)	10 (15.6)	26 (21.1)
Liver and/or hepatic (including gall bladder)	10 (20.8)	11 (17.2)	17 (13.8)
**PD-L1 status, n (%)**			
Positive (TC ≥1%)	22 (45.8)	31 (48.4)	47 (38.2)
1–49%	12 (25.0)	17 (26.6)	25 (20.3)
≥50%	10 (20.8)	14 (21.9)	22 (17.9)
Negative (TC <1%)	13 (27.1)	17 (26.6)	19 (15.4)
Unknown	13 (27.1)	16 (25.0)	57 (46.3)
**Prior regimens, n (%)**			
1	4 (8.3)	9 (14.1)	13 (10.6)
2	18 (37.5)	23 (35.9)	58 (47.2)
3	14 (29.2)	19 (29.7)	31 (25.2)
≥4	12 (25.0)	13 (20.3)	21 (17.1)
**Prior immunotherapies, n (%)**			
1	46 (95.8)	62 (96.9)	123 (100)
2	2 (4.2)	2 (3.1)	0
**Prior anti-PD-(L)1 immunotherapy, n (%)**			
Nivolumab	26 (54.2)	31 (48.4)	66 (53.7)
Pembrolizumab	16 (33.3)	23 (35.9)	34 (27.6)

Characteristic	Peripheral GEA	TCR analysis	
	Durvalumab-ceralasertib, *n* = 48	Durvalumab-ceralasertib, *n* = 64	Durvalumab plus olaparib or danvatirsen, *n* = 123
Atezolizumab	5 (10.4)	7 (10.9)	16 (13.0)
Durvalumab	1 (2.1)	1 (1.6)	6 (4.9)
Prior anti-CTLA4 immunotherapy, n (%)			
Tremelimumab	1 (2.1)	1 (1.6)	2 (1.6)
Ipilimumab	0	0	1 (0.8)
Best response on prior immunotherapy, n (%)			
Complete response	0	0	1 (0.8)
Partial response	12 (25.0)	14 (21.9)	35 (28.5)
Stable disease	26 (54.2)	33 (51.6)	34 (27.6)
Stable disease, biomarker-matched patients	9 (18.8)	11 (17.2)	13 (10.6)
Stable disease, biomarker-non-matched patients	17 (35.4)	22 (34.4)	21 (17.1)
Progressive disease	8 (16.7)	13 (20.3)	42 (34.1)
Non-evaluable	2 (4.2)	2 (3.1)	9 (7.3)
Not applicable	0	0	1 (0.8)
Time from prior immunotherapy	*n* = 48	*n* = 62	*n* = 122
Median, months (range)	4.2 (0.7–25.3)	3.4 (0.7–25.3)	3.0 (0.7–50.1)
Prior immunotherapy and resistance classification	*n* = 48		
Primary resistance, n (%)	18 (37.5)	25 (39.1)	56 (45.5)
Prior monotherapy	15 (31.3)	20 (31.3)	48 (39.0)
Prior combination	2 (4.2)	3 (4.7)	8 (6.5)
Both monotherapy and combination	1 (2.1)	2 (3.1)	0
Acquired resistance, n (%)	30 (62.5)	37 (57.8)	66 (53.7)
Prior monotherapy	26 (54.2)	32 (50.0)	52 (42.3)
Prior combination	3 (6.3)	4 (6.3)	13 (10.6)
Both monotherapy and combination	1 (2.1)	1 (1.6)	1 (0.8)
Not available, n (%)	0	2 (3.1)	1 (0.8)

Characteristic	Peripheral GEA	TCR analysis	
	**Durvalumab-ceralasertib, *n* = 48**	**Durvalumab-ceralasertib, *n* = 64**	**Durvalumab plus olaparib or danvatirsen, *n* = 123**
**Prior platinum-based therapies, n (%)**			
**1**	35 (72.9)	48 (75.0)	103 (83.7)
**2**	11 (22.9)	14 (21.9)	15 (12.2)
**≥3**	2 (4.2)	2 (3.1)	5 (4.1)
**Smoking status, n (%)**			
**Never**	7 (14.6)	7 (10.9)	23 (18.7)
**Current**	11 (22.9)	12 (18.8)	11 (8.9)
**Former**	30 (62.5)	45 (70.3)	89 (72.4)
**Neutrophil count, n (%)**			
**<10 x 10^9 cells/L**	44 (91.7)	59 (92.2)	110 (89.4)
**≥10 x 10^9 cells/L**	4 (8.3)	5 (7.8)	13 (10.6)
**Platelet count, n (%)**			
**<450 x 10^9 cells/L**	42 (87.5)	57 (89.1)	106 (86.2)
**≥450 x 10^9 cells/L**	6 (12.5)	7 (10.9)	17 (13.8)
**Haemoglobin level, n (%)**			
**>10 g/dL**	48 (100)	64 (100)	123 (100)
**≤10 g/dL**	0	0	0
**C-reactive protein, n (%)**			
**> overall median[e]**	22 (45.8)	29 (45.3)	61 (49.6)
**≤ overall median[e]**	26 (54.2)	35 (54.7)	59 (48.0)
**Lymphocyte count, n (%)**			
**> overall median[e]**	25 (52.1)	32 (50.0)	60 (48.8)
**≤ overall median[e]**	23 (47.9)	32 (50.0)	63 (51.2)
**Lactate dehydrogenase level, n (%)**			
**> overall median[e]**	24 (50.0)	30 (46.9)	60 (48.8)
**≤ overall median[e]**	22 (45.8)	32 (50.0)	61 (49.6)

[a]Sex as recorded by investigator in the case report form. [b]For parameters for which data are missing, the numbers of patients with data are indicated for each regimen and are used as the denominators for calculating percentages. [c]Recorded as 'Other' in the case report form. [d]Patient did not meet the eligibility criterion for ECOG PS of 0–1. [e]Overall median values for all patients within the respective study modules.

CNS, central nervous system; ECOG PS, Eastern Cooperative Oncology Group performance status; NOS, not otherwise specified; PD-L1, programmed cell death ligand 1.

Supplementary Figures

Supplementary Fig. 1 | Mutation profiles of patients who received durvalumab-ceralasertib by PFS on treatment. OncoPrint summaries of identified gene alterations for patients with available data enrolled to the **a**, ATM biomarker-matched cohort and **b**, biomarker-non-matched (ATM wild type) cohorts to receive durvalumab-ceralasertib, plus respective summary tables of individual gene alterations in patients with PFS <6 or ≥6 months within each cohort.

A

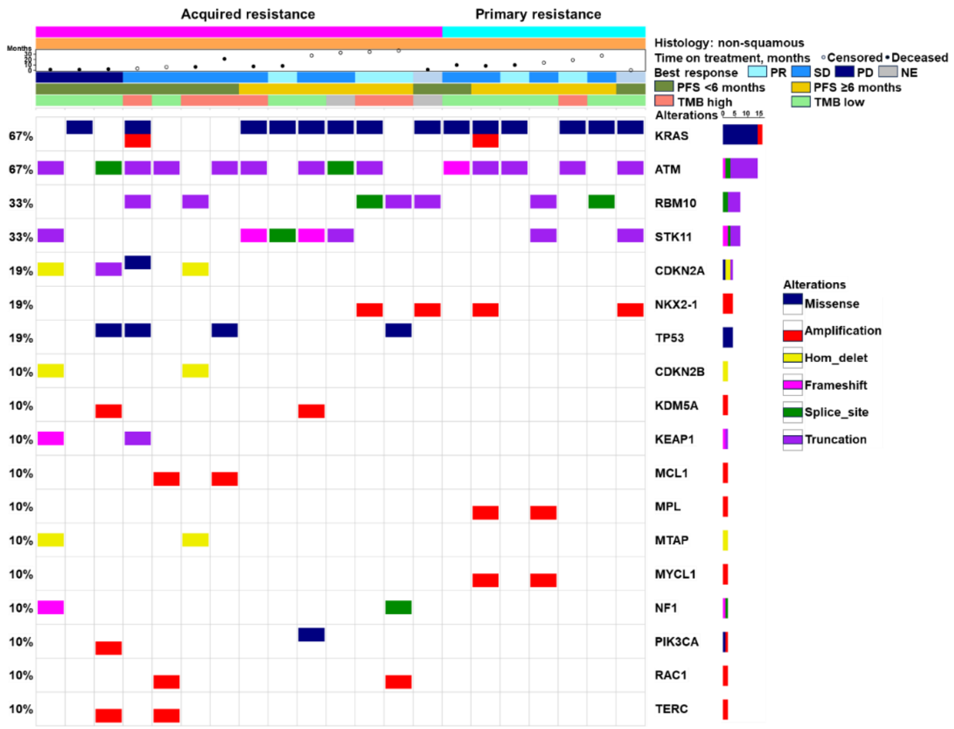

	Alteration frequency, n (%)	
**Gene**	**PFS <6 months (n=10)**	**PFS ≥6 months (n=11)**
**MPL**	**0**	**2 (18.2)**
**MYCL1**	**0**	**2 (18.2)**
**KRAS**	**5 (50.0)**	**9 (81.8)**
**STK11**	**2 (20.0)**	**5 (45.5)**
**RBM10**	**3 (30.0)**	**4 (36.4)**
**KDM5A**	**1 (10.0)**	**1 (9.1)**
**NF1**	**1 (10.0)**	**1 (9.1)**
**PIK3CA**	**1 (10.0)**	**1 (9.1)**
**RAC1**	**1 (10.0)**	**1 (9.1)**
**NKX2-1**	**2 (20.0)**	**2 (18.2)**
**ATM**	**7 (70.0)**	**7 (63.6)**
**TP53**	**3 (30.0)**	**1 (9.1)**
**CDKN2A**	**4 (40.0)**	**0**
**CDKN2B**	**2 (20.0)**	**0**
**KEAP1**	**2 (20.0)**	**0**
**MCL1**	**2 (20.0)**	**0**
**MTAP**	**2 (20.0)**	**0**
**TERC**	**2 (20.0)**	**0**

Table shows all genes with alterations seen in ≥2 patients (≥10%) in total, regardless of PFS. Top cells indicate a greater alteration frequency in in patients with PFS ≥6 months; bottom cells indicate a greater alteration frequency in patients with PFS <6 months. PFS, progression-free survival.

B

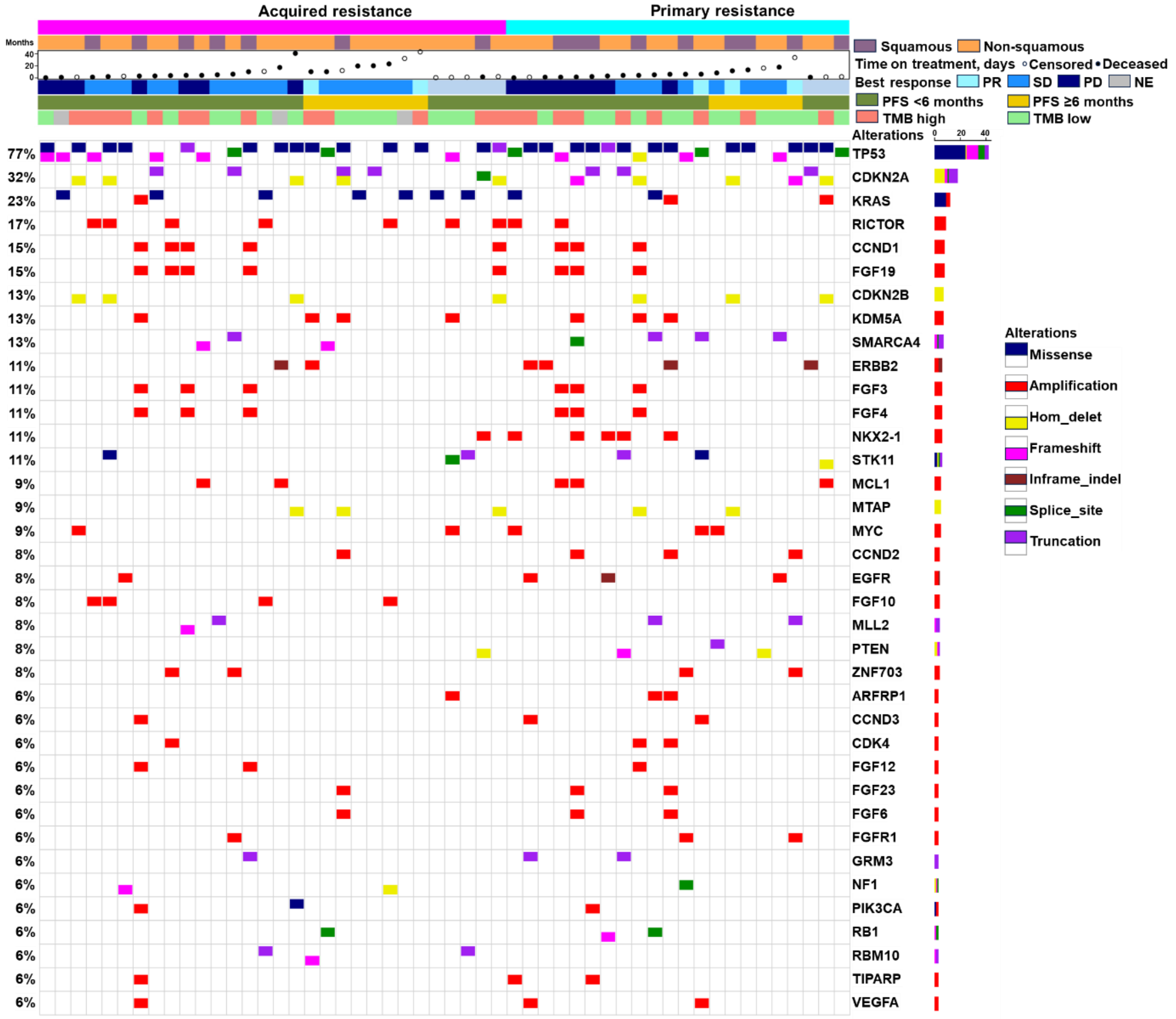

Gene	Alteration frequency, n (%) PFS <6 months (n=38)	PFS ≥6 months (n=14)
MTAP	3 (7.9)	2 (14.3)
KDM5A	5 (13.2)	2 (14.3)
SMARCA4	5 (13.2)	2 (14.3)
CDKN2A	13 (34.2)	4 (28.6)
MYC	4 (10.5)	1 (7.1)
ERBB2	5 (13.2)	1 (7.1)
KRAS	10 (26.3)	2 (14.3)
CDKN2B	6 (15.8)	1 (7.1)
TP53	32 (84.2)	9 (64.3)
RICTOR	8 (21.1)	1 (7.1)
CCND1	8 (21.1)	0
FGF19	8 (21.1)	0
FGF3	6 (15.8)	0
FGF4	6 (15.8)	0
NKX2-1	6 (15.8)	0
STK11	6 (15.8)	0
MCL1	5 (13.2)	0

Table shows all genes with alterations seen in ≥5 patients (≥10%) in total, regardless of PFS. Top cells indicate a greater alteration frequency in in patients with PFS ≥6 months; bottom cells indicate a greater alteration frequency in patients with PFS <6 months. PFS, progression-free survival.
