# Supplementary material for: Biomarker-directed targeted therapy plus durvalumab in advanced non-small-cell lung cancer: a phase 2 umbrella trial
Source: Nat Med. 2024 Feb 13;30(3):716–29. doi: 10.1038/s41591-024-02808-y (PMC10957481; doi:10.1038/s41591-024-02808-y)
Supplement: Supplementary file 2 — Reporting Summary [file 41591_2024_2808_MOESM2_ESM.pdf]

nature portfolio

Corresponding author(s): John V. Heymach

Last updated by author(s): Dec 11, 2023

Reporting Summary

Nature Portfolio wishes to improve the reproducibility of the work that we publish. This form provides structure for consistency and transparency in reporting. For further information on Nature Portfolio policies, see our Editorial Policies and the Editorial Policy Checklist.

Statistics

For all statistical analyses, confirm that the following items are present in the figure legend, table legend, main text, or Methods section.

n/a	Confirmed	
☐	☒	The exact sample size (*n*) for each experimental group/condition, given as a discrete number and unit of measurement
☐	☒	A statement on whether measurements were taken from distinct samples or whether the same sample was measured repeatedly
☐	☒	The statistical test(s) used AND whether they are one- or two-sided<br>*Only common tests should be described solely by name; describe more complex techniques in the Methods section.*
☐	☒	A description of all covariates tested
☐	☒	A description of any assumptions or corrections, such as tests of normality and adjustment for multiple comparisons
☐	☒	A full description of the statistical parameters including central tendency (e.g. means) or other basic estimates (e.g. regression coefficient) AND variation (e.g. standard deviation) or associated estimates of uncertainty (e.g. confidence intervals)
☐	☒	For null hypothesis testing, the test statistic (e.g. *F*, *t*, *r*) with confidence intervals, effect sizes, degrees of freedom and *P* value noted<br>*Give P values as exact values whenever suitable.*
☒	☐	For Bayesian analysis, information on the choice of priors and Markov chain Monte Carlo settings
☒	☐	For hierarchical and complex designs, identification of the appropriate level for tests and full reporting of outcomes
☒	☐	Estimates of effect sizes (e.g. Cohen's *d*, Pearson's *r*), indicating how they were calculated

Our web collection on statistics for biologists contains articles on many of the points above.

Software and code

Policy information about availability of computer code

Data collection	No software was used
Data analysis	SAS v9.3 for clinical data; R version 4.1.0 for violin plots and heatmaps (Fig 2, Ext Data Fig 1); GSVA R package version 1.42.0 for enrichment scores; nSolver 4.0 for gene expression; R version 4.2 for TCR data (Fig 5)

For manuscripts utilizing custom algorithms or software that are central to the research but not yet described in published literature, software must be made available to editors and reviewers. We strongly encourage code deposition in a community repository (e.g. GitHub). See the Nature Portfolio guidelines for submitting code & software for further information.

Data

Policy information about availability of data

All manuscripts must include a data availability statement. This statement should provide the following information, where applicable:

- Accession codes, unique identifiers, or web links for publicly available datasets
- A description of any restrictions on data availability
- For clinical datasets or third party data, please ensure that the statement adheres to our policy

Data underlying the findings described in this manuscript may be obtained in accordance with AstraZeneca's data sharing policy described at https://astrazenecagrouptrials.pharmacm.com/ST/Submission/Disclosure. Data for studies directly listed on Vivli can be requested through Vivli at www.vivli.org. Data for

studies not listed on Vivli could be requested through Vivli at https://vivli.org/members/enquiries-about-studies-not-listed-on-the-vivli-platform/. AstraZeneca's Vivli member page is also available outlining further details: https://vivli.org/ourmember/astrazeneca/.

Research involving human participants, their data, or biological material

Policy information about studies with human participants or human data. See also policy information about sex, gender (identity/presentation), and sexual orientation and race, ethnicity and racism.

Reporting on sex and gender	Data on sex were collected via the Case Report Form and are reported in the patient baseline characteristics table. Analyses reported in this paper are not controlled for sex. Preliminary analyses from early on in the HUDSON study showed a complete overlap in PFS data and in OS data between male and female patients, and so no further sex-based analyses were conducted.
Reporting on race, ethnicity, or other socially relevant groupings	Data on race were collected via the Case Report Form using standard reporting categories and reported in the patient baseline characteristics tables. Analyses were not controlled for race or ethnicity
Population characteristics	Patients with advanced NSCLC who have received a platinum-doublet regimen and progressed on anti-PD-(L)1-based therapy; median age 63-64 years.
Recruitment	Patients were recruited by participating cancer treatment centres. Patients being treated by the centres who required a new line of treatment were considered for study eligibility; patients who then signed informed pre-screening consent were considered 'enrolled' patients per protocol. Each enrolled patient was identified by a unique E-code. Enrolled patients underwent a biopsy to determine biomarker specification of their tumours, and provided information regarding the study eligibility criteria, overall and for specific individual modules, via a computer system. Patients were then allocated to a treatment cohort based on their molecular profile via an web-based Interactive Response System (IRT), and assigned an additional unique patient number prior to first dose. There was no potential for self-selection bias or other biases.
Ethics oversight	The study is being performed in accordance with consensus ethical principles derived from international guidelines including the Declaration of Helsinki and Council for International Organizations of Medical Sciences (CIOMS) International Ethical Guidelines, applicable International Council for Harmonisation (ICH) Good Clinical Practice (GCP) Guidelines, all applicable laws and regulations, and the AstraZeneca policy on Bioethics and Human Biological Samples. The study protocol, protocol amendments, informed consent form, investigator's brochure, and other relevant documents were reviewed and approved by the Institutional Review Board/Ethics Committee at each participating centre: Austria – Wiener Gesundheitsverbund–Klinik Floridsdorf; Uniklinikum Salzburg; LBI for Lung Health, c/o Klinik Penzing, Wiener Gesundheitsverbund; Universitätsklinik Innsbruck. Canada – Princess Margaret Cancer Centre; William Osler Health System–Brampton Civic Hospital; Alberta Health Services; The Ottawa Hospital; Centre Hospitalier de l'Universite de Montreal. France – CHU Nantes Hôpital Laennec; Institut Gustave Roussy; Institut Bergonié; Hôpital Bichat–Claude-Bernard. Israel – Rabin Medical Center–Beilinson Hospital; Rambam Health Care Campus; Meir Hospital. South Korea – Samsung Medical Center; Seoul National University Hospital; Asan Medical Center. Spain – Hospital Universitario Ramón y Cajal; Hospital Universitario Virgen Macarena. United States – The University of Texas; Johns Hopkins University; UCLA; Washington University in St. Louis School of Medicine; St Joseph Heritage Healthcare; Dana Farber Mass General Brigham Cancer Care Inc; City of Hope National Medical Center; Fox Chase Cancer Center; Sarah Cannon Research Institute at Tennessee Oncology; New York-Presbyterian; University of California San Diego; Sibley Memorial Hospital; Virginia Cancer Specialists; University of Pittsburgh School of Medicine.

Note that full information on the approval of the study protocol must also be provided in the manuscript.

Field-specific reporting

Please select the one below that is the best fit for your research. If you are not sure, read the appropriate sections before making your selection.

☒ Life sciences ☐ Behavioural & social sciences ☐ Ecological, evolutionary & environmental sciences

For a reference copy of the document with all sections, see nature.com/documents/nr-reporting-summary-flat.pdf

Life sciences study design

All studies must disclose on these points even when the disclosure is negative.

Sample size	Initial sample size for each cohort was to be 20 evaluable patients, with possible expansion based on observation of an efficacy signal per the primary endpoint of ORR. Evaluations were to be carried out after the ~20th evaluable patient in a cohort, or the final patient dosed in a cohort if enrolment ended early, had had the opportunity for two on-treatment response assessments per RECIST or had discontinued or withdrawn from treatment. A statistical framework was built around a prespecified ORR to inform the Sponsor if an efficacy signal had been observed and a recommendation to expand should be made. Decisions regarding stopping recruitment in specific cohorts were at the discretion of the Sponsor and were based on emerging efficacy, safety, and tolerability data.
Data exclusions	No data were excluded.
Replication	No replication was performed as this was a clinical trial. The objective was to obtain an assessment of the efficacy of each treatment by evaluation of objective response rate for each study module/treatment. There was no within-study comparison and replication was limited to treatment across multiple patients within a study arm (n=20).

Randomization	No randomization was performed. Although this was a non-comparative study, covariates are important to the evaluation of data from this study. Specific biomarkers were used to assign patients to modules in Group A (biomarker-matched), and for non-biomarker matched patients (Group B), cohorts were stratified into patients who were primary resistant or developed acquired resistance while on immunotherapy. Further stratification by covariates was not feasible due to the small patient numbers within each module.
Blinding	No blinding was performed as this was an open-label, non-randomised, non-comparative study evaluating multiple different regimens and dosing schedules within multiple biomarker-matched and biomarker-unmatched cohorts.

Reporting for specific materials, systems and methods

We require information from authors about some types of materials, experimental systems and methods used in many studies. Here, indicate whether each material, system or method listed is relevant to your study. If you are not sure if a list item applies to your research, read the appropriate section before selecting a response.

Materials & experimental systems

n/a	Involved in the study
☒	☐ Antibodies
☒	☐ Eukaryotic cell lines
☒	☐ Palaeontology and archaeology
☒	☐ Animals and other organisms
☐	☒ Clinical data
☒	☐ Dual use research of concern
☒	☐ Plants

Methods

n/a	Involved in the study
☒	☐ ChIP-seq
☒	☐ Flow cytometry
☒	☐ MRI-based neuroimaging

Clinical data

Policy information about clinical studies

All manuscripts should comply with the ICMJE guidelines for publication of clinical research and a completed CONSORT checklist must be included with all submissions.

Clinical trial registration	NCT03334617
Study protocol	ClinicalTrials.gov - full protocol not yet available for this ongoing pharmaceutical company-sponsored trial
Data collection	Between January 26, 2018, and April 26, 2022, 941 patients were screened for enrolment in HUDSON, including 324 for the four modules reported herein; of these, 268 patients received treatment at the participating centres: Austria – Wiener Gesundheitsverbund–Klinik Floridsdorf; Uniklinikum Salzburg; LBI for Lung Health, c/o Klinik Penzing, Wiener Gesundheitsverbund; Universitätsklinik Innsbruck. Canada – Princess Margaret Cancer Centre; William Osler Health System–Brampton Civic Hospital; Alberta Health Services; The Ottawa Hospital; Centre Hospitalier de l'Universite de Montreal. France – CHU Nantes Hôpital Laennec; Institut Gustave Roussy; Institut Bergonié; Hôpital Bichat–Claude-Bernard. Israel – Rabin Medical Center–Beilinson Hospital; Rambam Health Care Campus; Meir Hospital. South Korea – Samsung Medical Center; Seoul National University Hospital; Asan Medical Center. Spain – Hospital Universitario Ramón y Cajal; Hospital Universitario Virgen Macarena. United States – The University of Texas; Johns Hopkins University; UCLA; Washington University in St. Louis School of Medicine; St Joseph Heritage Healthcare; Dana Farber Mass General Brigham Cancer Care Inc; City of Hope National Medical Center; Fox Chase Cancer Center; Sarah Cannon Research Institute at Tennessee Oncology; New York-Presbyterian; University of California San Diego; Sibley Memorial Hospital; Virginia Cancer Specialists; University of Pittsburgh School of Medicine. Data collection was via a computer system by which study sites provided data for review by the Sponsor.
Outcomes	The primary objective is to assess the objective response rate (ORR) for each treatment combination according to Response Evaluation Criteria for Solid Tumours (RECIST) version 1.1. Secondary objectives include the assessment of disease control rate, progression-free survival (PFS), and overall survival (OS), as well as the safety and tolerability of each treatment regimen. Tumours were assessed using contrast enhanced computed tomography (CT; or magnetic resonance imaging [MRI] if CT was contraindicated) scans of the chest, abdomen, and pelvis (including liver and adrenal glands) at baseline, every 6 weeks (±1 week) for the first 24 weeks from the start of combination therapy, and every 8 weeks (±1 week) thereafter until disease progression (confirmed with a subsequent scan). Patients who, in the opinion of the investigator, were still receiving clinical benefit from treatment, were permitted to continue treatment following confirmed radiological disease progression per RECIST v1.1. Scheduled radiological scans were continued for these patients while they were still receiving study treatment.<br>For the primary outcome of ORR, objective tumour responses were assessed and categorised programmatically per RECIST v1.1 based on the scans described above, interpreted according to RECIST 1.1. Survival status was recorded every 3 months (±1 week) after a safety follow-up visit (90 days after study drug discontinuation) and until the earlier of: 12 months after the last patient had discontinued treatment in the last cohort within a module; or 75% of patients had died in all cohorts within a module. Safety was assessed and AEs recorded throughout the treatment period and the safety follow-up (90 days after the discontinuation of all study drugs or until initiation of another therapy, unless the investigator assesses that the event occurring within 90 days after last dose of study treatment but after the initiation of another therapy is related to the study treatment). Severity of AEs was assessed per the National Cancer Institute's Common Terminology Criteria for Adverse Events (NCI CTCAE) version 4.03. Causal relationship between study drug and each AE was assessed by the investigators, by answering 'yes' or 'no' to the question: 'Do you consider that there is a reasonable possibility that the event may have been caused by the study drug?'.
